# Pridopidine in early-stage manifest Huntington’s disease: a phase 3 trial

**DOI:** 10.1038/s41591-025-03920-3

**Published:** 2025-09-05

**Authors:** Ralf Reilmann, Andrew Feigin, Anne E. Rosser, Sandra K. Kostyk, Carsten Saft, Yael Cohen, Henk Schuring, Randal Hand, Andrew M. Tan, Kelly Chen, Wei Feng, Leehee Navon-Perry, Andres Cruz-Herranz, Christine Syltevik, Diderik Boot, Ferdinando Squitieri, Elise Kayson, Munish Mehra, Y. Paul Goldberg, Michal Geva, Michael R. Hayden

**Affiliations:** 1 George Huntington Institute (GHI), Muenster, Germany; 2 NYU Langone Health, New York, NY USA; 3 Brain Repair Group, Cardiff University, Cardiff, UK; 4 Advanced Neurotherapeutics Centre, Neuroscience and Mental Health Innovation Institute, Division of Psychological Medicine and Clinical Neurosciences, Cardiff University, Cardiff, UK; 5 Departments of Neurology and Neuroscience, The Ohio State University Wexner Medical Center, Columbus, OH USA; 6 Department of Neurology, Huntington-Centre NRW, St. Josef Hospital, Ruhr-University Bochum, Bochum, Germany; 7 Prilenia Therapeutics B.V., Naarden, the Netherlands; 8 Department of Neurology, Yale University School of Medicine, New Haven, CT USA; 9 Unità Huntington e Malattie Rare, Fondazione IRCCS Casa Sollievo della Sofferenza, San Giovanni Rotondo, Italy; 10 Huntington Study Group, Rochester, NY USA; 11 Quantum BioPharma, Gaithersburg, MD USA; 12 Department of Medical Genetics, Centre for Molecular Medicine and Therapeutics, University of British Columbia, Vancouver, British Columbia Canada

**Keywords:** Drug development, Huntington's disease

## Abstract

Huntington’s disease (HD) is a rare, neurodegenerative disorder for which only symptomatic treatments are available. The PROOF-HD study was a randomized, double-blind, placebo-controlled phase 3 trial evaluating the efficacy and safety of pridopidine, a selective Sigma-1 receptor agonist, in HD. The primary and key secondary endpoints, change in total functional capacity (TFC) and composite Unified Huntington’s Disease Rating Scale (cUHDRS) score at week 65, were not met in the overall population. The TFC least-squares mean difference between pridopidine and placebo was −0.18 (95% confidence interval −0.49 to 0.14; *P* = 0.26). The cUHDRS least-squares mean difference between pridopidine and placebo was −0.11 (95% confidence interval −0.40 to 0.18; *P* = 0.45). Sensitivity analysis in a subgroup of participants not treated with antidopaminergic medications at any time demonstrated a consistent pattern favoring pridopidine across multiple measures, including TFC and cUHDRS. Notably, pridopidine 45 mg twice daily demonstrated a favorable safety and tolerability profile. Taken together, pridopidine has the potential to address a critical unmet need in HD. ClinicalTrials.gov identifier: NCT04556656.

## Abstract (web summary)

In a phase 3 trial for Huntington’s disease, pridopidine did not meet its primary endpoints but was safe and favored in some prespecified subgroup analyses.

## Main

Huntington’s disease (HD) is a rare autosomal dominant disorder caused by a genetic mutation in the *huntingtin* gene. HD progresses over 10–20 years, causing motor abnormalities, cognitive decline, psychiatric symptoms and progressive loss of independence that ultimately result in death^1–3^. Symptomatic treatments exist, but therapies to modify disease progression are urgently needed^2,4,5^. Standard treatments for HD include antidopaminergic medications (ADMs) such as vesicular monoamine transporter 2 inhibitors (VMAT2 inhibitors) for chorea and off-label antipsychotics (neuroleptics), which may help reduce involuntary movements and behavioral symptoms^4–7^. However, mounting evidence suggests ADM-related side effects may interfere with accurate measures of disease progression, especially in outcomes like cognition and function widely used in HD clinical trials^5,8–10^.

Pridopidine is a potent and selective Sigma-1 receptor (S1R) agonist and potential new HD therapy. By targeting S1R, pridopidine modulates multiple pathways in HD and other neurodegenerative diseases^11^. Preclinical evidence demonstrates that pridopidine exerts neuroprotective effects through a multimodal action—reducing endoplasmic reticulum stress, promoting calcium homeostasis and stimulating neurotrophic-dependent restoration of synaptic plasticity^12–18^. A positron emission tomography study in humans confirms pridopidine’s engagement with S1R in key HD-affected brain regions, including the striatum^19^. Clinical trials show oral pridopidine is well-tolerated with a benign safety profile^20–22^ and may have the potential to preserve functional capacity, cognition and motor function in HD^20–26^.

In early clinical studies, including the HART and MermaiHD trials^21,22,26,27^, motor-related endpoints were primary outcomes but were not met. However, exploratory analyses indicated pridopidine 45 mg twice daily (bid) showed motor benefits with significant improvements in total motor score (TMS). In PRIDE-HD^23,24^, pridopidine 45 mg bid was associated with a slower rate of decline in total functional capacity (TFC), a key measure of functional abilities in HD, over 52 weeks compared with placebo, with potential benefits lasting up to five years in the Open-HART extension study^20,21^. In the phase 2 HEALEY ALS Platform Trial, pridopidine 45 mg bid showed numerical trends suggesting slowed decline in ALSFRS-R, respiratory, bulbar and speech functions in a post hoc analysis of a subgroup of participants meeting El-Escorial Criteria for definite or probable amyotrophic lateral sclerosis within 18 months of symptom onset^28^.

These findings supported further evaluation of pridopidine in PROOF-HD (Pridopidine-Outcome-on-Function), a global phase 3, randomized, double-blind, placebo-controlled trial in early adult-onset HD. This study (ClinicalTrials.gov identifier: NCT04556656; EudraCT number 2020-002822-10; date of registration 16 October 2020) assessed the efficacy and safety of pridopidine 45 mg bid on clinical measures of HD progression. The primary endpoint was the change in TFC from baseline to week 65 (ref. ^3^). A key secondary endpoint was the change in composite Unified Huntington’s Disease Rating Scale (cUHDRS), a sensitive global measure of function, cognition and motor assessments^29^.

To reflect clinical practice and given the duration of the study (18 months), the trial permitted the concomitant use of ADMs, which are standard treatments for psychiatric symptoms and chorea in HD. However, given their known side effects, such as parkinsonism, sedation and cognitive impairment, which may confound trial outcomes^5,8,9,30^, a sensitivity analysis was conducted in a subgroup of participants who remained off ADMs (no VMAT2 inhibitors or antipsychotics at any time during the study)^31^. This allowed examination of pridopidine’s potential treatment effect in the absence of possible ADM-related confounding^30,31^.

In the overall population, pridopidine did not significantly outperform placebo in the primary or key secondary endpoints. However, in a sensitivity analysis of the off-ADMs subgroup, pridopidine was associated with favorable trends across measures of function, cognition, motor performance and cUHDRS scores. These findings underscore the importance of minimizing potential ADM-related confounding in HD trials and support further investigation of pridopidine in defined patient subgroups.

## Results

### Participant disposition

**Fig. 1:**
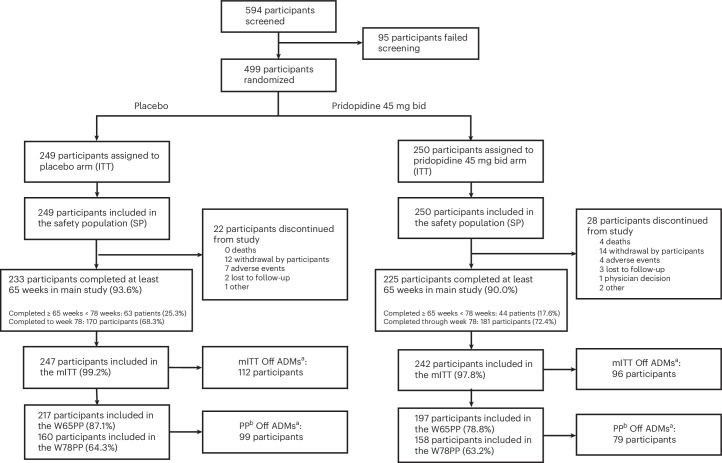
Participant disposition flowchart. The flowchart illustrates participant disposition across study populations. ‘Off ADMs’ refers to participants who were not treated with ADMs at any point during the study. The ITT population includes all randomized participants. The mITT population consists of ITT participants who received at least one dose of the study drug and had valid TFC scores at baseline and at least one post-baseline visit. The SP population includes randomized participants who received at least one dose of the study drug. The PP population represents the combined analysis set of the W65PP and W78PP subsets. The W65PP and W78PP subsets include mITT participants with valid TFC scores at weeks 65 and 78, at least 80% compliance with the study drug and no major protocol deviations. W65PP, per protocol week 65 population; W78PP, per protocol week 78 population. ^a^Patients who did not receive ADM treatment at any point during the study; ^b^the analysis set consisting of patients from both W65PP and W78PP.

Between October 2020 and March 2023, 594 participants were screened across 59 international sites. Of these, 499 were randomized 1:1 to receive either pridopidine 45 mg bid or placebo (intent-to-treat (ITT): placebo, *n* = 249; pridopidine, *n* = 250) (Fig. 1). The modified intent-to-treat (mITT) population excluded ten participants because of missing post-baseline efficacy data (mITT: placebo, *n* = 247; pridopidine, *n* = 242). Completion rates at week 65 were 90.0% (pridopidine) and 93.6% (placebo), and at week 78 were 72.4% and 68.3%, respectively, under the common closing study design (Methods). The per protocol (PP) population included 217 participants in the placebo group and 197 in the pridopidine group at week 65, and 160 in the placebo group and 158 in the pridopidine group at week 78. Twenty-eight participants in the pridopidine group and 22 in the placebo group discontinued for various reasons (Fig. 1 and Extended Data Table 1).

Baseline demographic and disease characteristics were comparable across populations. In the ITT population, the mean (s.d.) age was 52.5 (11.7) years, CAG repeat length was 43.9 (3.54) and 51.9% of participants were female. The disease stage distribution showed 41.3% of participants were at stage HD1 (TFC 11–13) and 58.7% were at stage HD2 (TFC 7–10). The mITT group showed similar distributions (Table 1). Randomization was stratified by baseline HD stage and antipsychotic use. VMAT2 inhibitor use, which was not a stratification factor, was by chance slightly higher in the pridopidine group (22.3%) versus placebo (15.4%).

**Table 1:** Participant demographics and baseline disease characteristics

Parameter	mITT		mITT off ADMs^g^ any time during the study		PP^h^ off ADMs^g^ any time during the study	
	Placebo (*n* = 247)	Pridopidine (*n* = 242)	Placebo (*n* = 112)	Pridopidine (*n* = 96)	Placebo (*n* = 99)	Pridopidine (*n* = 79)
Age (years), mean (s.d.)	52.6 (11.37)	52.4 (11.92)	52.3 (11.28)	50.9 (11.02)	51.9 (11.20)	50.9 (10.81)
BMI (kg m^−2^), mean (s.d.)	25.21 (4.778)	24.98 (5.02)	24.6 (4.39)	25 (4.89)	24.5 (4.44)	25.1 (5.07)
Female, *n* (%)	126 (51.0)	129 (53.3)	62 (55.4)	54 (56.3)	56 (56.6)	48 (60.8)
Male, *n* (%)	121 (49.0)	113 (46.7)	50 (44.6)	42 (43.8)	43 (43.4)	31 (39.2)
Duration since onset of symptoms (years), mean (s.d.)	4.63 (4.586)	4.36 (3.26)	4.1 (4.02)	3.9 (3.15)	4 (4.02)	3.8 (2.78)
HD1 (TFC 11–13)	102 (41.3)	100 (41.3)	57 (50.9)	46 (47.9)	51 (51.5)	39 (49.4)
HD2 (TFC 7–10)	145 (58.7)	142 (58.7)	55 (49.1)	50 (52.1)	48 (48.5)	40 (50.6)
CAG repeat length, mean (s.d.)	43.6 (3.28)	44.1 (3.78)	43.1 (2.98)	44.0 (3.45)	43.2 (2.94)	44.0 (3.24)
CAP^a^, mean (s.d.)	494.4 (92.76)	512.8 (83.33)	467.6 (86.63)	497.0 (81.08)	472.2 (84.65)	500.6 (81.32)
UHDRS-IS score^b^, mean (s.d.)	81.4 (6.64)	81.9 (6.53)	83.3 (6.10)	83.0 (6.09)	83.5 (6.12)	83.4 (5.81)
CAP100^i^, mean (s.d.)	105.84 (15.420)	108.55 (13.859)	101.52 (15.304)	105.27 (13.654)	102.05 (15.181)	105.86 (13.701)
TFC score, mean (s.d.)	9.9 (1.71)	9.9 (1.69)	10.3 (1.60)	10.2 (1.59)	10.3 (1.62)	10.3 (1.52)
UHDRS-TMS score^c^, mean (s.d.)	32.9 (10.95)	33.8 (11.16)	30.3 (9.64)	31.3 (11.05)	30.2 (9.98)	30.8 (11.23)
SDMT score, mean (s.d.)	23.3 (9.31)	22.8 (9.02)	26.7 (9.42)	25.7 (8.96)	26.2 (9.29)	26.1 (9.31)
SWR score, mean (s.d.)	62.0 (18.15)	61.0 (17.70)	68.9 (16.40)	64.6 (17.56)	68.4 (15.47)	65.5 (17.30)
cUHDRS score^d^, mean (s.d.)	8.9 (2.65)	8.7 (2.53)	9.9 (2.42)	9.5 (2.50)	9.8 (2.43)	9.6 (2.47)
HD-QoL score^e^, mean (s.d.)	60.8 (37.68)	61.6 (40.54)	53.5 (34.44)	54.0 (39.92)	55.6 (34.64)	53.1 (41.57)
CGI-S score^f^, mean (s.d.)	3.2 (0.79)	3.3 (0.81)	3.1 (0.85)	3.1 (0.85)	3.1 (0.82)	3.1 (0.83)
FT IOI, average, both hands (ms), mean (s.d.)	353.3 (137.78)	352.9 (129.92)	329.5 (95.29)	352.4 (112.24)	333.4 (95.74)	353 (111.41)

The table summarizes participant demographics and baseline disease characteristics across the ITT, mITT and PP populations. The ITT population includes all randomized participants, the mITT population includes those with valid baseline and post-baseline TFC scores, and the PP population represents the combined analysis set of W65PP and W78PP. At baseline, mean age, CAG repeat length and gender distribution were similar across treatment arms. ADM use was 44.5% in the placebo group and 50.0% in the pridopidine group, with antipsychotics and VMAT2 inhibitors as the most common ADMs. During the study, 45.3% of participants receiving placebo and 39.7% of participants receiving pridopidine remained off ADMs. ‘Off ADMs’ refers to participants who did not receive ADM treatment during the study. Stage HD1 and HD2 distributions were similar across off-ADM subgroups.

^a^CAP = Age × (CAG − 33.66).

^b^UHDRS-IS, Unified Huntington’s Disease Rating Scale Independence Scale; scores collected at screening.

^c^UHDRS-TMS, Unified Huntington’s Disease Rating Scale-Total Motor Score.

^d^cUHDRS = [(TFC − 10.4)/1.9] − [(TMS − 29.7)/14.9] + [(SDMT − 28.4)/11.3] + [(SWR − 66.1)/20.1] + 10.

^e^HD-QoL, Huntington’s Disease-Quality of Life.

^f^CGI-S, The Clinical Global Impression-Severity

^g^ADM, Antidopaminergic Medication.

^h^PP, Per Protocol population.

^i^CAP100, participants with a CAG-Age Product (CAP) score of 100.

ADM use was permitted post-baseline, if deemed necessary by the treating physician. At baseline, 44.5% (110 of 247) of participants in the placebo population and 50.0% (121 of 242) of participants in the pridopidine mITT population used ADMs. Antipsychotics (a stratification factor) were used by 33.2% (placebo) and 31.8% (pridopidine) of participants; VMAT2 inhibitors were used by 15.4% (placebo) and 22.7% (pridopidine) of participants. During the study, 45.3% (placebo) and 39.7% (pridopidine) of participants remained off ADMs. Baseline HD stage among mITT and PP off-ADM participants was balanced: ~50% in HD1 and HD2 (Table 1).

### Primary outcome

#### Change in TFC at week 65

**Fig. 2:**
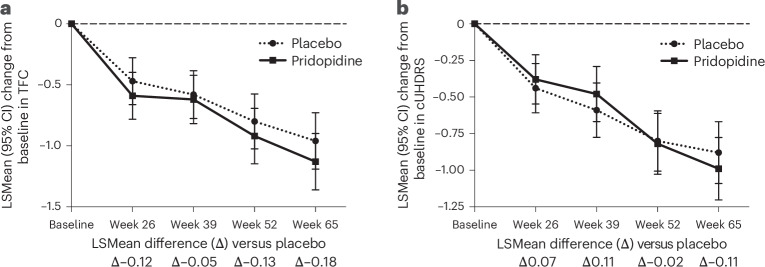
Primary and key secondary endpoints in the overall population. **a**,**b**, TFC (primary endpoint) (**a**) and cUHDRS (key secondary endpoint) (**b**) are presented as LSMean changes from baseline to week 65 for the ITT and mITT populations. LSMean differences between the placebo and pridopidine treatment arms are shown at each time point. Sample sizes at week 65 were: placebo, *n* = 249 and pridopidine, *n* = 250 (ITT); and placebo, *n* = 247 and pridopidine, *n* = 242 (mITT). All replicates represent biologically independent participants. Statistical analyses were performed using a maximum likelihood-based mixed-effects model for repeated measures (MMRM). Data are presented as LSMean ± 95% CI.

The primary outcome was the change in TFC from baseline to week 65. In the ITT population (placebo, *n* = 249; pridopidine, *n* = 250), pridopidine did not outperform placebo (Fig. 2a). The least-squares mean (LSMean) change was −0.95 (95% confidence interval (CI) −1.18 to −0.72) for placebo and −1.12 (95% CI −1.36 to −0.89) for pridopidine; difference −0.18 (95% CI −0.49 to 0.14; *P* = 0.27) (Fig. 2a and Extended Data Table 2).

### Key secondary outcome

#### Change in composite UHDRS at week 65

The multiplicity-adjusted key secondary endpoint in the overall population was the change in cUHDRS from baseline at week 65. No significant difference in cUHDRS was observed at week 65 (mITT: LSMean difference −0.11 (95% CI −0.40 to 0.18); *P* = 0.454). The change in cUHDRS was −0.88 (95% CI −1.09 to −0.66) for placebo and −0.99 (95% CI −1.20 to −0.77) for pridopidine (Fig. 2b and Extended Data Table 3).

### Safety and tolerability

Pridopidine 45 mg bid was well-tolerated, with a safety profile broadly comparable with placebo^22,23,26^, and consistent with previous clinical trial experience (Table 2 and Extended Data Table 4). Compliance in the safety population (SP) was 94.5%, with an average treatment duration of 70.7 weeks. Treatment-emergent adverse events (TEAEs) occurred in 85.9% of participants in the placebo population and 82.8% of participants the pridopidine population, with >70% of TEAEs classified as mild or moderate. Common TEAEs included COVID-19, falls, diarrhea and headache. Depression was more frequent in the pridopidine group (10.4% versus 5.2%) but was below historical HD norms^32–34^. Suicidality rates were comparable between groups (0.4% in each).

**Table 2:** Treatment-emergent adverse events and serious adverse events

	Placebo (*n*, %) (*N* = 249)	Pridopidine (*n*, %) (*N* = 250)	Total (*n*, %) (*N* = 499)
TEAEs			
COVID-19	58 (23.3)	60 (24.0)	118 (23.6)
Fall	58 (23.3)	55 (22.0)	113 (22.6)
Diarrhea	22 (8.8)	21 (8.4)	43 (8.6)
Headache	25 (10.0)	16 (6.4)	41 (8.2)
Depression	13 (5.2)	26 (10.4)	39 (7.8)
Insomnia	18 (7.2)	20 (8.0)	38 (7.6)
Anxiety	17 (6.8)	20 (8.0)	37 (7.4)
Nasopharyngitis	18 (7.2)	19 (7.6)	37 (7.4)
Urinary tract infection	17 (6.8)	11 (4.4)	28 (5.6)
Back pain	14 (5.6)	13 (5.2)	27 (5.4)
Contusion	15 (6.0)	12 (4.8)	27 (5.4)
SAEs			
Total number of SAEs	21 (8.4)	34 (13.6)	55 (11.0)
Psychiatric disorders	6 (2.4)	10 (4.0)	16 (3.2)
Injury, poisoning and procedural complications	5 (2.0)	4 (1.6)	9 (1.8)
Infections and infestations	2 (0.8)	4 (1.6)	6 (1.2)
Neoplasms benign, malignant and unspecified (including cysts and polyps)	1 (0.4)	5 (2.0)	6 (1.2)
Gastrointestinal disorders	1 (0.4)	4 (1.6)	5 (1.0)
Nervous system disorders	2 (0.8)	3 (1.2)	5 (1.0)
Cardiac disorders	3 (1.2)	1 (0.4)	4 (0.8)
Musculoskeletal and connective tissue disorders	1 (0.4)	2 (0.8)	3 (0.6)
General disorders and administration site conditions	0	2 (0.8)	2 (0.4)
Metabolism and nutrition disorders	1 (0.4)	1 (0.4)	2 (0.4)
Blood and lymphatic system disorders	1 (0.4)	0	1 (0.2)
Hepatobiliary disorders	0	1 (0.4)	1 (0.2)
Investigations	0	1 (0.4)	1 (0.2)
Respiratory, thoracic and mediastinal disorders	0	1 (0.4)	1 (0.2)
Vascular disorders	0	1 (0.4)	1 (0.2)

Summary of the most common TEAEs, occurring in ≥5% of participants in either treatment group, and all SAEs reported in the placebo and pridopidine 45 mg bid groups. TEAEs are listed by preferred terms in decreasing order of frequency, with COVID-19, falls and diarrhea being the most commonly reported events. SAEs are categorized by system organ class, with psychiatric disorders (for example, depression, anxiety) and injury-related complications being the most frequently reported. Other notable SAEs included neoplasms (benign, malignant and unspecified), infections, gastrointestinal disorders and nervous system disorders.

Serious adverse events (SAEs) were reported in 11% of participants (8.4% placebo, 13.6% pridopidine), with none assessed as related to study treatment. No safety signal indicating increased mortality risk with pridopidine treatment was identified. Of the four deaths reported during the trial, none was considered related to the study drug (one in the placebo arm and three in the pridopidine arm) (Extended Data Table 1). The single death in the placebo group followed a motor vehicle accident. In the pridopidine group, the deaths were attributed to clinical complications consistent with the natural progression of HD^35^. The only treatment-related SAE (acute myocardial infarction) was in the placebo group. Psychiatric SAEs were infrequent and generally balanced across groups, including suicidal ideation (two in each group) and isolated cases of anxiety, delusion and depression. Suicide attempts occurred in three participants in the pridopidine group, all of whom had pre-existing psychiatric comorbidities. These events are consistent with the elevated background risk of suicidality in HD^36^.

Treatment-related TEAEs led to discontinuation in 0.8% of participants in the placebo group and 0.4% in the pridopidine group (Extended Data Table 1). Severe treatment-related TEAEs were rare (0.4% placebo, 1.2% pridopidine), with no treatment-related serious events in the pridopidine group and no evidence of a broader safety signal, as reported previously^35^. Pridopidine caused no clinically meaningful changes in renal function or Q-T interval duration. Q-T interval corrected using Fridericia’s formula (Q-TcF) changes >30 ms but ≤60 ms occurred in 9.2% of participants in the pridopidine group and 3.6% of in the placebo group, with no post-baseline Q-TcF values >480 ms or change >60 ms, indicating no cardiac safety concerns.

### Sensitivity analyses in participants off ADMs

**Fig. 3:**
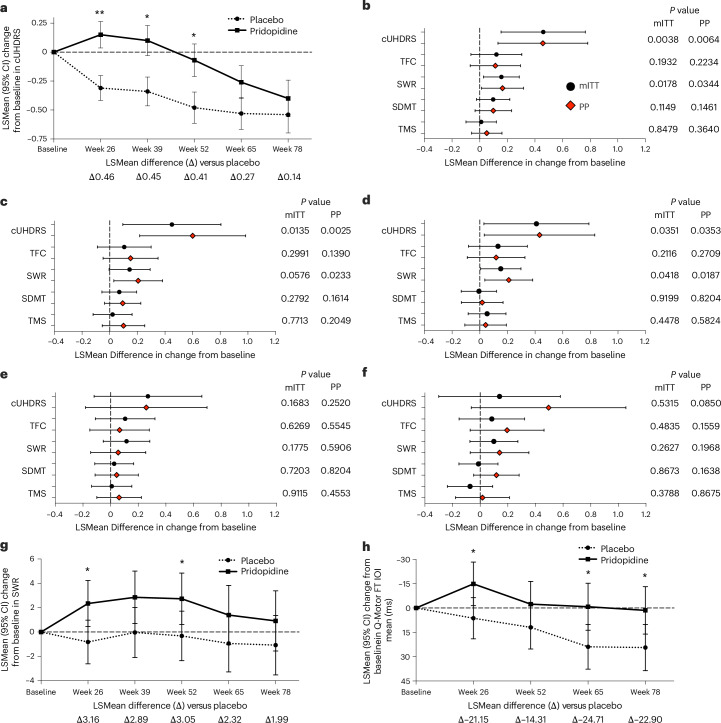
Sensitivity analysis of clinical outcomes in the off-ADM subgroup. **a**, In the off-ADM mITT population, pridopidine treatment was associated with a slower rate of decline in cUHDRS scores compared with placebo. Maximal differences were observed between weeks 26 and 52, with nominal statistical significance at weeks 26 (*P* = 0.004), 39 (*P* = 0.014) and 52 (*P* = 0.035). **b**–**f**, Forest plots display treatment effects for cUHDRS and its four components—TFC, SWR, SDMT and TMS—in both the off-ADM mITT and PP populations. For the mITT group, sample sizes were as follows: week 26, *n* = 112 (placebo) and *n* = 95 (pridopidine) (**b**); week 39, *n* = 107 (placebo) and *n* = 90 (pridopidine) (**c**); week 52, *n* = 104 (placebo) and *n* = 91 (pridopidine) (**d**); week 65, *n* = 106 (placebo) and *n* = 91 (pridopidine) (**e**); and week 78, *n* = 71 (placebo) and *n* = 70 (pridopidine) (**f**). For the PP population, group sizes were: week 26, *n* = 99 (placebo) and *n* = 78 (pridopidine) (**b**); week 39, *n* = 99 (placebo) and *n* = 76 (pridopidine) (**c**); week 52, *n* = 97 (placebo) and *n* = 78 (pridopidine) (**d**); week 65, *n* = 97 (placebo) and *n* = 78 (pridopidine) (**e**); and week 78, *n* = 60 (placebo) and *n* = 60 (pridopidine) (**f**). **g**, Cognitive performance based on SWR showed numerical improvements with pridopidine at week 26 (*P* = 0.018; *n* = 112 (placebo) and *n* = 95 (pridopidine)), week 39 (*P* = 0.058; *n* = 107 (placebo) and *n* = 91 (pridopidine)) and week 52 (*P* = 0.042; *n* = 104 (placebo) and *n* = 92 (pridopidine)). **h**, Motor skills assessed by FT IOI Mean showed significant improvements at week 26 (*P* = 0.025; *n* = 112 (placebo) and *n* = 95 (pridopidine)), week 65 (*P* = 0.016; *n* = 104 (placebo) and *n* = 91 (pridopidine)) and week 78 (*P* = 0.028; *n* = 71 (placebo) and *n* = 69 (pridopidine)). All replicates represent independent participants. Statistical analyses were performed using a MMRM with two-sided nominal *P* values. The unit of analysis was the individual participant. Data are presented as LSMean, with error bars indicating 95% CI. *P < 0.05, **P < 0.01.

**Extended Data Fig. 1:**
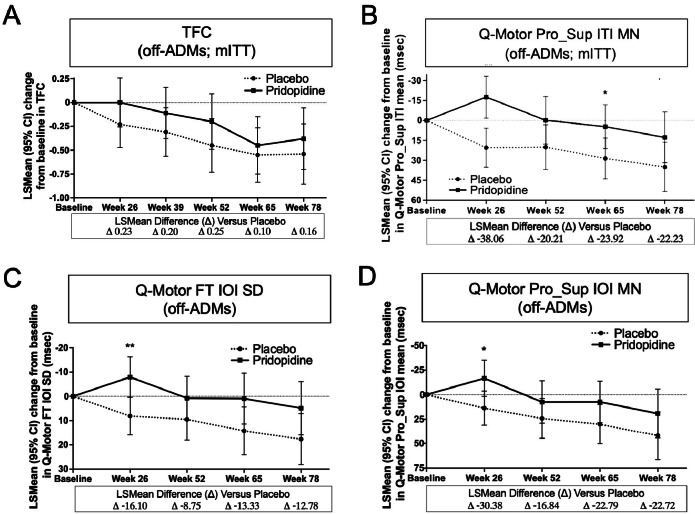
Sensitivity Analysis in mITT Participants Off ADMs Showing the Effect of Pridopidine on TFC, cUHDRS, Cognitive Function (SWR) and Motor Performance (Q-Motor Assessments). *Sensitivity analyses* of the change in (**a**) TFC show that pridopidine treatment showed a numerical trend favoring pridopidine, slowing TFC decline as compared with placebo through Week 78 with maximal TFC difference at Week 52 (difference: 0.26 vs. placebo; n = 104 placebo, n = 91 pridopidine). (**b**) *Skilled Motor Performance (Pronation-Supination Inter-Tap Interval [Pro-Sup ITI] Mean):* Improvements were significant at Week 26 (LSMean difference = -38.06 msec; p = 0.007; n = 112 placebo, n = 95 pridopidine) and Week 65 (LSMean difference = -23.92 msec; p = 0.04), with numerical trends favoring pridopidine at other timepoints. (**c**, **d**) Pridopidine treatment mitigated declines in motor function in mITT participants off ADMs. Negative values indicate improvement. Specifically: (**c**) *Motor Variability (Finger Tapping IOI Standard Deviation [SD]):* Significant reductions in variability were observed at Week 26 (LSMean difference = -0.43; p = 0.035; n = 112 placebo, n = 95 pridopidine), with trends favoring pridopidine at later timepoints. (**d**) *Pronation-Supination Inter-Onset Interval (Pro-Sup IOI) Mean:* Significant improvements were observed at Week 26 (LSMean difference = -17.2 msec; p = 0.044; n = 112 placebo, n = 95 pridopidine). Each panel represents mean change from baseline with 95% confidence intervals. *p < 0.05; **p < 0.01; ***p < 0.001. All statistical tests are based on mixed-effects models for repeated measures (MMRM) applied to the mITT population off ADMs. Negative values indicate improvement for all motor outcomes.

**Extended Data Fig. 2:**
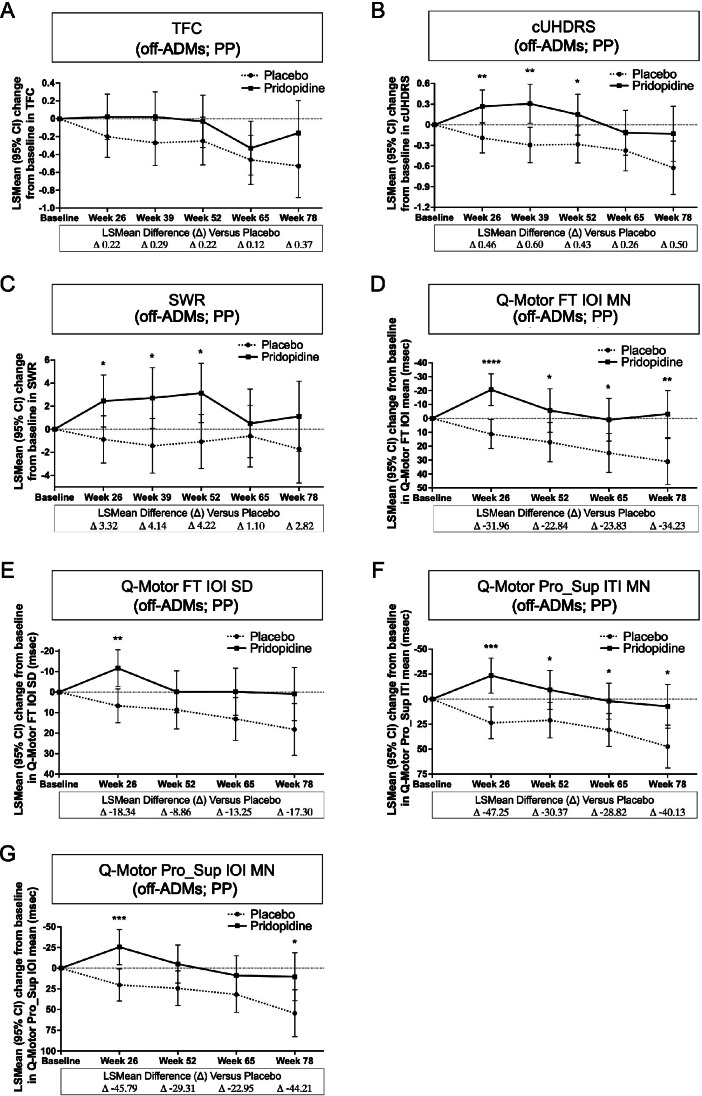
Sensitivity Analysis in PP Participants Off ADMs Showing the Effect of Pridopidine on TFC, cUHDRS, Cognitive Function (SWR) and Motor Performance (Q-Motor Assessments). (**a**) Pridopidine showed a 0.37-point difference in TFC versus placebo at Week 78 (placebo: n = 160; pridopidine: n = 158). (**b**) For the cUHDRS, pridopidine slowed decline compared with placebo, with nominal statistical significance observed through Week 52 (Week 26: p = 0.006, placebo: n = 99, pridopidine: n = 78; Week 39: p = 0.003, n = 99, n = 76; Week 52: p = 0.035, n = 97, n = 78) (*p < 0.05; **p < 0.01). (**c**) Pridopidine demonstrated improvements in cognitive function (SWR) with significance reached at Week 26 (p = 0.034, n = 99, n = 78), Week 39 (p = 0.023, n = 99, n = 77), and Week 52 (p = 0.019, n = 97, n = 79) (*p < 0.05; **p < 0.01). (**d**–**g**) For motor outcomes, negative values indicate improvement. (**d**) Motor Skills (Finger Tapping Inter-Onset Interval [FT IOI]): Pridopidine resulted in significant improvements at Week 26 (p < 0.0001, n = 99, n = 78), with sustained differences through Week 78 (n = 76, n = 74; *p < 0.05; **p < 0.01; ****p < 0.0001). (**e**) Motor Variability (FT IOI Standard Deviation [SD]): Significant reductions were observed at Week 26 (p < 0.01; n = 99, n = 78) (**p < 0.01). (**f**) Skilled Motor Performance (Pronation-Supination Inter-Tap Interval [Pro-Sup ITI]): Improvements were significant at Week 26 (***p < 0.001), with trends favoring pridopidine through Week 78 (*p < 0.05; **p < 0.01; n = 99, n = 78). (**g**) Pronation-Supination Inter-Onset Interval (Pro-Sup IOI): Significant improvements were observed at Week 26 (**p < 0.01; n = 99, n = 78) and Week 78 (*p < 0.05; n = 76, n = 74). All data represent mean change from baseline in the PP population off-ADMs. Statistical significance was assessed using mixed-effects models for repeated measures (MMRM). P values are nominal and unadjusted for multiplicity. Error bars represent 95% confidence intervals (CIs).

A subgroup analysis evaluated pridopidine’s effects in participants not treated with ADMs, which may confound measures of clinical outcome^30,31^. Because the primary endpoint was not met, all analyses for the off-ADM subgroup report *P* values as nominal and unadjusted for multiplicity. Figure 3 and Extended Data Figs. 1 and 2 present outcomes from the off-ADM subgroup analyses in the mITT and PP populations, respectively, with summaries below.

#### Impact of pridopidine on functional decline (TFC)

In the mITT population off ADMs, pridopidine demonstrated a trend favoring slower TFC decline compared with placebo to week 78, with the strongest effect at week 52 (maximal difference: 0.25 versus placebo) (Extended Data Fig. 1a). Confidence intervals overlapped at all time points. Whereas pridopidine showed a trend for slower decline, participants in the placebo group showed the expected pattern of progressive TFC decline. In the PP population, pridopidine showed a showed a numerically favorable trend (a 0.37-point difference versus placebo at week 78), but this was not significant (Extended Data Fig. 2a).

#### Impact of pridopidine on global clinical progression (cUHDRS)

In the mITT population off ADMs, pridopidine showed nominally significant benefits in maintaining cUHDRS scores compared with placebo, with the largest differences (Δ) at week 26 (Δ = 0.46, *P* = 0.004; placebo *n* = 112, pridopidine *n* = 95), week 39 (Δ = 0.45, *P* = 0.014; placebo *n* = 107, pridopidine *n* = 90) and week 52 (Δ = 0.41, *P* = 0.035; placebo *n* = 104, pridopidine *n* = 91) (Fig. 3a). Improvements from baseline were most pronounced to week 39. Differences at week 65 (Δ = 0.27, *P* = 0.168; placebo *n* = 106, pridopidine *n* = 91) and week 78 (Δ = 0.14, *P* = 0.532; placebo *n* = 71, pridopidine *n* = 70) were not significant. In the PP population off-ADMs, similar treatment effects were also observed at week 26 (Δ = 0.46, *P* = 0.006; placebo *n* = 99, pridopidine *n* = 78), week 39 (Δ = 0.60, *P* = 0.003; placebo *n* = 99, pridopidine *n* = 76) and week 52 (Δ = 0.43, *P* = 0.035; placebo *n* = 97, pridopidine *n* = 78), with nonsignificant trends at weeks 65 and 78 (Extended Data Fig. 2b).

A key assumption in using the multicomponent cUHDRS is that therapies targeting disease progression should lead to improvements across multiple components of the cUHDRS^29,37^. To evaluate this, a forest plot analysis assessed the contributions of TFC, Stroop Word Reading test (SWR), Symbol Digital Modality Test (SDMT) and TMS among participants off-ADMs (Fig. 3b–f). The analysis showed that differences favoring pridopidine were evident across all four cUHDRS components, rather than being driven by any single one. TFC and SWR showed trends favoring pridopidine at all time points. Although TFC did not reach significance, point estimates favored pridopidine up to week 78 in both the mITT and PP populations. For SWR, differences were observed at week 26 (mITT *P* = 0.018; PP *P* = 0.034), week 39 (mITT *P* = 0.0576; PP *P* = 0.023) and week 52 (mITT *P* = 0.042; PP *P* = 0.019), indicating potential cognitive benefit with pridopidine (Fig. 3b–d). SDMT and TMS did not show differences, but TMS trended in favor of pridopidine at weeks 26, 39 and 52. These findings suggest that the observed cUHDRS treatment effect differences in the off-ADM subgroup were supported by contributions from multiple outcomes, across function, cognition and motor measures.

#### Effect of pridopidine on cognitive function

In the mITT population off ADMs, pridopidine was associated with a consistent pattern of slowing cognitive decline compared with placebo to week 52. For SWR, between-group differences favored pridopidine across multiple time points (Fig. 3b–g), with LSMean differences of 3.16 at week 26 (*P* = 0.018; placebo *n* = 112, pridopidine *n* = 95), 2.89 at week 39 (*P* = 0.058; placebo *n* = 107, pridopidine *n* = 91) and 3.05 at week 52 (*P* = 0.042; placebo *n* = 104, pridopidine *n* = 92) (Fig. 3g). For SDMT, trends also favored pridopidine to week 39, with score trajectories remaining stable to week 78 (Fig. 3b–g). In the PP population off ADMs, a similar trend was observed, with significant SWR differences at week 26 (*P* = 0.034; placebo *n* = 99, pridopidine *n* = 78), 4.14 at week 39 (*P* = 0.023; placebo *n* = 99, pridopidine *n* = 77) and 4.22 at week 52 (*P* = 0.019; placebo *n* = 97, pridopidine *n* = 79) (Extended Data Fig. 2c).

#### Effect of pridopidine on motor function

Motor outcomes in participants in the off-ADM subgroup showed a pattern of numerical improvements with pridopidine treatment, based on Q-Motor measures including the finger tapping inter-onset-interval (FT IOI) (Fig. 3h). In these measures, pridopidine treatment resulted in improved FT IOI motor performance. At week 26, the LSMean difference in FT IOI was −21.15 ms (*P* = 0.025; placebo *n* = 112, pridopidine *n* = 95), with additional numerical differences favoring pridopidine at week 52 (−14.31 ms, *P* = 0.15; placebo *n* = 104, pridopidine *n* = 91), week 65 (−24.71 ms, *P* = 0.016; placebo *n* = 104, pridopidine *n* = 91) and week 78 (−22.90 ms, *P* = 0.028; placebo *n* = 71, pridopidine *n* = 69).

For Q-Motor task pronation–supination tapping inter-tap-interval (Pro-Sup ITI), pridopidine improved function at weeks 26 and 65 compared with placebo (Extended Data Fig. 1b). At week 26, the LSMean difference was −38.06 ms (*P* = 0.007; placebo *n* = 112, pridopidine *n* = 95) and at week 65, the LSMean difference was −23.92 ms (*P* = 0.04; placebo *n* = 104, pridopidine *n* = 91). Although improvements at week 52 (−20.21 ms, *P* = 0.109) and week 78 (−22.23 ms, *P* = 0.104) did not reach statistical significance, the overall trend favored pridopidine.

In the PP population, these effects in FT IOI and Pro-Sup ITI were more pronounced, with larger LSMean differences observed throughout the study. Results consistently favored pridopidine over placebo at time points between weeks 26 and 78, supporting a potential treatment effect on motor performance (Extended Data Fig. 2d,f).

Additional variability data from finger tapping (FT IOI s.d.) (Extended Data Fig. 1c) also showed a beneficial effect of pridopidine at week 26, with a nominally significant improvement over placebo. At later time points, weeks 52, 65 and 78, the treatment effect persisted as a numerical trend in favor of pridopidine. In the PP population, results were more robust, with larger treatment effects observed consistently across all time points (Extended Data Fig. 2e).

Results for Pro-Sup IOI show similar trends favoring pridopidine, particularly at weeks 26 and 65 (Extended Data Fig. 1d). Effects were more pronounced in the PP population (Extended Data Fig. 2g). Together, although these motor findings suggest pridopidine’s potential to improve motor function and mitigate motor decline in HD, these outcomes were secondary, and should therefore be interpreted cautiously.

### Post hoc analyses

#### Responder and threshold analyses of cUHDRS scores in participants off ADMs

To further characterize the treatment effect in the off-ADM subgroup, two post hoc analyses of cUHDRS scores were conducted to explore clinically meaningful response patterns. These analyses provided a complementary view to mean change estimates, highlighting potential differences in individual participant trajectories in a population that typically experiences progressive decline in HD^2,29,38^ (Methods).

**Fig. 4:**
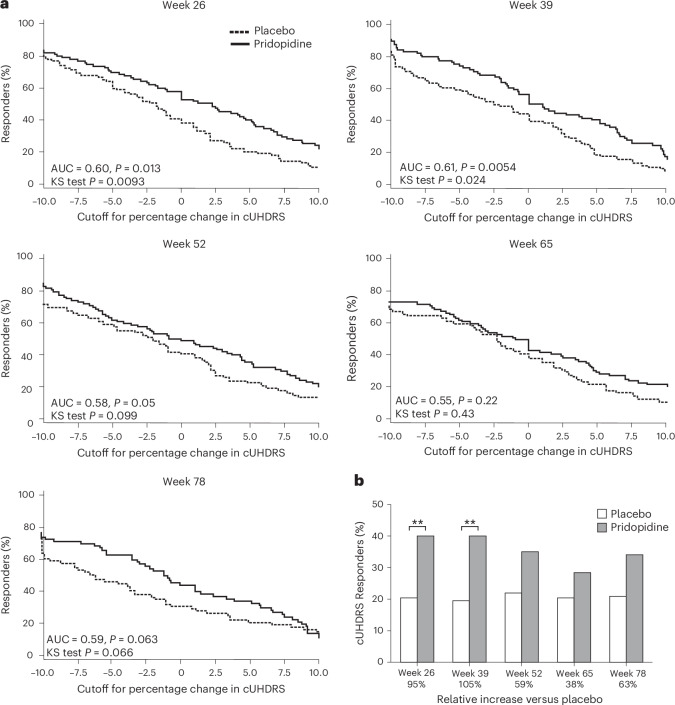
Responder and threshold analyses of cUHDRS scores in participants off ADMs. **a**, Post hoc responder analyses in the mITT population show that pridopidine led to higher CDF response rates than placebo at all time points, with significant differences at weeks 26 (AUC = 0.60, *P* = 0.013; KS test *P* = 0.0093) and 39 (AUC = 0.61, *P* = 0.0054; KS test *P* = 0.024). **b**, A threshold-based analysis revealed that a ≥5% improvement in cUHDRS was achieved in approximately twice as many participants receiving pridopidine compared with placebo at weeks 26 (*P* = 0.004) and 39 (*P* = 0.003). All statistical tests were two-sided and unadjusted for multiple comparisons. Error bars represent 95% CI. **P < 0.01.

**Extended Data Fig. 3:**
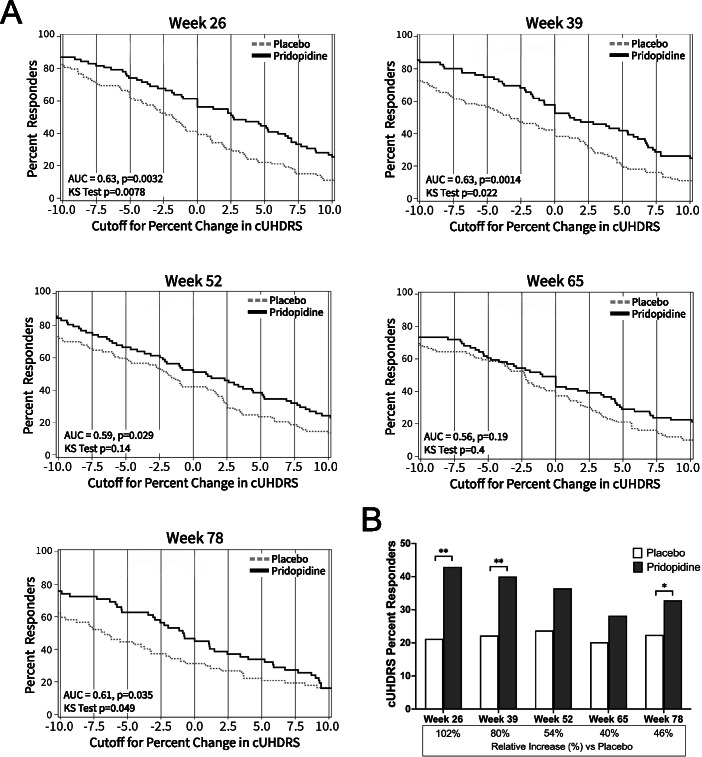
*Post Hoc* Cumulative Distribution Function Analysis Based on Percent Change from Baseline in Composite Unified Huntington’s Disease Rating Scale Through Week 78 in Participants Off Antidopaminergic Medications (PP). (**a**) This *post hoc* analysis presents the cumulative distribution function (CDF) of percent change from baseline in cUHDRS scores through Week 78 in the PP population of participants not receiving antidopaminergic medications (ADMs). Pridopidine consistently demonstrated higher response rates compared with placebo, with significant differences observed at Week 26 (AUC = 0.63, p = 0.003; KS Test p = 0.008; placebo n = 99, pridopidine n = 78) and Week 39 (AUC = 0.63, p = 0.001; KS Test p = 0.02; n = 99, n = 76). Trends favoring pridopidine persisted at Week 65 (AUC = 0.56; n = 97, n = 78) and Week 78 (AUC = 0.61; n = 60, n = 60). These results highlight pridopidine’s potential to improve or maintain function in participants off ADMs, with pronounced effects observed during the first 39 weeks. (**b**) A threshold-based analysis revealed that a ≥ 5% improvement in cUHDRS was achieved in approximately twice as many participants receiving pridopidine compared with placebo at Week 26 (p = 0.006) and Week 39 (p = 0.004) (**p < 0.01; *p < 0.05). All statistical tests were two-sided and unadjusted for multiple comparisons. Error bars represent 95% confidence intervals (CIs). AUC = area under the curve; KS test = Kolmogorov–Smirnov test.

The first approach used cumulative distribution functions (CDFs) in the mITT population and showed consistently greater response rates for pridopidine than placebo across all time points (Fig. 4a). The CDF provides a comprehensive view of the distribution of treatment responses without relying on a predefined cutoff, thereby reducing potential bias and allowing for objective comparison of clinical benefit across groups. At week 26, the treatment difference was notable (area under the curve (AUC) = 0.60, *P* = 0.013; Kolmogorov–Smirnov (KS) test *P* = 0.0093), with similar trends at weeks 39 (AUC = 0.61), 65 (AUC = 0.55) and 78 (AUC = 0.59). In the PP population, effects were more pronounced at week 78 (*P* = 0.035) (Extended Data Fig. 3).

In the second approach, a post hoc threshold-based analysis using a ≥5% improvement cutoff in cUHDRS score consistently showed higher response rates for pridopidine compared with placebo across all time points (Fig. 4b and Methods). At weeks 26 and 39, response rates nearly doubled for pridopidine (week 26: 40% versus 21%, *P* = 0.004; week 39: 40% versus 20%, *P* = 0.003). This corresponds to a relative increase in responders of 95% at week 26 and 104% at week 39. This pattern persisted to week 78, suggesting potential durability. The PP population showed similar findings, with significant differences observed at weeks 26, 39 and 78. Together, these responder analyses provide additional insight into a potential treatment effect of pridopidine in participants off ADMs, particularly with favorable trends sustained to week 78.

## Discussion

The PROOF-HD study was a multicenter, randomized, double-blind, placebo-controlled phase 3 trial evaluating the efficacy and safety of pridopidine over 65 weeks, with a variable 13-week extension for a total of 78 weeks. The trial evaluated pridopidine’s impact on functional, motor and cognitive decline in participants with HD. Completion rates were high, with 91.8% of participants completing the 65-week main study. These findings underscore the feasibility and robustness of long-term pridopidine treatment. In the overall population, the study did not meet its primary endpoint of change in TFC from baseline to week 65, or the key secondary endpoint of change in cUHDRS.

Several factors may inform interpretation and underscore the limitations of this trial. The trial was conducted during the COVID-19 pandemic, which impacted global healthcare access. As observed in other neuropsychiatric trials, a higher-than-expected use of ADMs was observed^39–41^. This may have introduced confounding, because many ADMs are associated with side effects such as sedation, parkinsonism and cognitive impairment that are well-documented in regulatory labels^42–45^ and can mimic or mask HD symptoms^8,30,31^. In addition to these symptomatic effects, the complex relationship between prolonged D2 antagonism from ADMs and the risk of extrapyramidal symptoms (EPS) varies among participants^46–48^; further complicating the interpretation of treatment effects in trials like PROOF-HD, where symptomatic overlap may obscure true drug-related benefits.

Given that many ADMs are metabolized by cytochrome P450 2D6 (refs. ^42–45,49,50^) and that pridopidine inhibits cytochrome P450 2D6, it is also possible that pharmacokinetic interactions in the active treatment arm could have further interfered with measures of clinical outcome in the overall population^51^. These limitations suggest that ADM-related confounding may reduce the sensitivity of functional and cognitive measures in HD trials, and support the need for future studies to stratify randomization by ADM use or to prespecify subgroup analyses based on ADM exposure status. Although this study was not designed to test dosage strategies for ADMs, findings also raise the possibility that ADM dose minimization (as recommended in regulatory labeling) or careful ADM and dosage selection in combination with pridopidine may offer a viable strategy for symptom management^20,24,31^.

To assess the potential effect of pridopidine treatment in the absence of ADM interference, we performed a sensitivity analysis of an off-ADM subgroup, which excluded participants who received VMAT2 inhibitors or antipsychotics at any time during the trial. Although no significant difference with treatment was observed in the off-ADM cohort for TFC, this cohort was smaller than anticipated, and consequently may have limited statistical power. By contrast, nominally favorable trends were observed across multiple other outcomes, including in cUHDRS, TFC, SWR and Q-Motor^23,24,52^, particularly baseline to week 52, suggesting the potential for slowed progression relative to placebo over 1 year. These findings were based on nominal *P* values without correction for multiplicity and should be interpreted with caution. Here, participants off ADMs treated with pridopidine may have experienced numerically smaller declines in cUHDRS scores over 78 weeks. Note that modest annual reductions in cUHDRS (0.1–0.3 points) have been associated with a clinical meaningful benefit in HD^29^, underscoring the potential importance of these trends. In complementary post hoc responder analyses of cUHDRS, data further suggested higher response rates for pridopidine compared with placebo over time^53^. Improvements in SWR, reflecting cognitive flexibility and processing speed were also observed at early time points, consistent with possible cognitive benefit in a disease with relentless decline.

Objective Q-Motor results also provided rater-independent insight of motor function, aligning with clinical outcomes^23,54^. Compared with placebo, nominally significant improvements were seen in FT IOI (weeks 26 and 78) and Pro-Sup ITI (weeks 26 and 65). FT IOI, in particular, has been shown to detect motor impairments up to 20 years before clinical onset in HD gene carriers and to track longitudinal progression across disease stages^23,55^. It demonstrates a strong structure–function relationship, correlating with HD-related caudate and putamen atrophy as well as clinical progression in outcomes such as TMS and TFC^2,23,55^. These motor outcomes paralleled trends in cUHDRS and support the internal consistency of treatment effects across clinical measures, although these are secondary endpoints and require cautious interpretation.

Overall, participants treated with pridopidine in the off-ADM subgroup showed less decline than participants treated with placebo across multiple outcomes, but the subgroup size was limited, and the findings were not powered to support definitive conclusions. Nonetheless, the consistency of trends across function, motor and cognition suggests a treatment effect. To date, no approved therapy has demonstrated a definitive, consistent effect on global progression endpoints like TFC or cUHDRS in HD. Although preliminary, observed trends with pridopidine treatment highlight pridopidine’s potential to address HD progression beyond symptom-specific treatments like VMAT2 inhibitors for chorea.

Consistent with earlier trials like PRIDE-HD^21–23^, pridopidine was well-tolerated, and with a safety profile comparable to placebo. High study completion (91.8% for 65 weeks) and long treatment duration support its feasibility for long-term use; an essential component for continued research and development efforts^20,23,25^. Open-label data from Open-HART also support the long-term safety of pridopidine^20^, with no treatment-related deaths or cardiac risk signals identified in PROOF-HD. Rates of psychiatric events and suicidality were in line with background HD risk, and no new safety concerns emerged^6,25,35^.

Pridopidine’s therapeutic effects are closely tied to its potent and highly selective S1R agonist activity^56^. S1R is a chaperone protein involved in cellular stress adaptation, neuroprotection and synaptic function^57^. Activation of S1R by pridopidine has been shown to reduce endoplasmic reticulum stress, support calcium homeostasis and promote neurotrophic signaling^14,16,58,59^. These effects are particularly relevant to HD pathophysiology, which involves progressive striatal degeneration and impaired cellular resilience.

In conclusion, pridopidine did not demonstrate significant benefit in the overall PROOF-HD population. However, the consistent safety profile, pharmacologic rationale and the coherence of treatment effects across multiple outcomes in the off-ADM sensitivity analysis—findings not previously observed in any HD trial—identify a biologically plausible and clinically relevant patient population in which treatment benefit was observed. This merits further evaluation in confirmatory trials. Future studies should refine patient selection and account for the potential impact of ADM exposure through stratification and dosage strategies, which may preserve pridopidine’s effects while also managing symptoms.

## Methods

### Trial design

This phase 3, randomized, double-blind, placebo-controlled, parallel-arm, multicenter clinical trial (PROOF-HD; ClinicalTrials.gov ID NCT04556656, EudraCT number 2020-002822-10) aimed to evaluate the efficacy and safety of pridopidine at a dose of 45 mg bid in participants with a TFC score range of 7–13 at screening. Recruitment took place across 59 international sites in Austria, Canada, Czechia, France, Germany, Italy, Netherlands, Poland, Spain, the UK and the USA. The trial was conducted primarily during the global COVID-19 pandemic, with the first participant enrolled on 23 October 2020, and the last patient completing the double-blinded portion of the study on 14 March 2023.

The study enrolled 499 participants, and participants were randomized in a 1:1 ratio to receive either pridopidine or placebo (ITT population *n* = 249 placebo and *n* = 250 pridopidine). Randomization was stratified based on baseline HD stage (HD1 TFC 11–13 versus HD2 TFC 7–10) and antipsychotic use at baseline (yes or no). The randomization sequence was generated by the Sponsor’s designated statistical team, and allocation was concealed from site investigators, participants and study staff throughout the trial. The study consisted of a screening period, randomization and a double-blind treatment period. The study was conducted over a period of 65–78 weeks, with the primary analysis taking place at 65 weeks to assess primary efficacy, key secondary and other outcomes, as well as safety. An additional variable 13-week double-blind treatment period was included to evaluate the durability of the treatment effect up to 78 weeks.

Following the baseline visit, participants underwent a 2-week titration period of 45 mg once daily, followed by 63 weeks of full-dose maintenance treatment (45 mg bid or matching placebo). Participants in the active arm received pridopidine at the clinically recommended dose of 45 mg bid orally administered as hard gelatin capsules, whereas those in the placebo arm received indistinguishable matching placebo capsules. Participants who completed the 65-week maintenance period entered a variable double-blind extension period of up to 13 weeks, concluding when the last randomized participant completed 65 weeks of treatment. In this common closing design, all participants remaining between weeks 65 and 78 completed ‘end of study’ visits once the final participant reached week 65. This approach allowed for the collection of extended double-blind, controlled data to evaluate efficacy up to 78 weeks, while ensuring consistent double-blinding for all participants and investigators throughout the study.

Most participants were followed for 78 weeks, including the mITT population of 490 participants (98.2%), which comprised those who received at least one dose of the study drug and had valid baseline and post-baseline assessments. Of all participants, 458 (91.8%) completed at least 65 weeks in the main study period. There were two PP populations: one for week 65 and one for week 78. The week 65 per protocol (W65PP) population comprised 414 patients (83.0% of the ITT) and included participants from the mITT group who had valid TFC data at week 65, maintained >80% compliance with the study drug and had no substantial protocol deviations impacting TFC assessment. The week 78 per protocol (W78PP) population comprised 318 participants (63.7% of the ITT) and was defined similar to W65PP but included participants with valid TFC data at week 78.

The dose selection of 45 mg bid for pridopidine in the PROOF-HD study was based on evidence from previous research, notably the phase 2 PRIDE-HD study^19,23,24^. This study demonstrated the potential efficacy of pridopidine at this dose, particularly in early-stage HD patients. The 45 mg bid dose aimed to optimize efficacy while maintaining an acceptable safety profile, as observed in the PRIDE-HD study. The PRIDE-HD study also highlighted pridopidine’s potential to maintain functional capacity in participants with TFC scores ranging from 7 to 13 over a 52-week period, with the most significant benefits observed in those classified as HD1 or HD2. Considering that individuals with HD1 and HD2 (TFC 7–13) typically experience an annual decline in TFC of roughly 0.8 to 1.0 points, the PROOF-HD phase 3 study was designed with a 65-week observation period^24,29^. This extended timeframe was chosen to allow sufficient opportunity to detect a potential maintenance effect of pridopidine compared to the natural decline anticipated in the placebo arm.

Sex and gender were self-reported by participants at screening and documented in the electronic case report forms. The study was not stratified by sex or gender, and no subgroup analyses were conducted for this study based on these characteristics. Participants received financial compensation as outlined in site contracts for each in-person clinic visit, as well as for telephone, virtual or home-based visits. Additional reimbursement for transportation or lodging costs was considered on a case-by-case basis with prior approval by the study contract research organization (CRO).

### Trial eligibility criteria

Inclusion and exclusion criteria were established to enroll participants with early to moderate HD, defined by motor, functional and genetic parameters. These criteria prioritized participant safety and facilitated the creation of a homogeneous study population, enabling accurate assessment of pridopidine’s effects. Detailed protocol information is available on ClinicalTrials.gov (accessed 23 January 2025; https://clinicaltrials.gov/study/NCT04556656).

#### Inclusion criteria

Participants had to meet all of the following criteria:

- Age ≥25 years at the time of signing informed consent
- Male or female
- Diagnosis of HD based on clinical features and the presence of ≥36 CAG repeats in the *huntingtin* (*HTT*) gene, confirmed historically or at screening
- Diagnostic confidence level of 4 (≥99% certainty) on the UHDRS-TMS
- Adult-onset HD (onset of signs or symptoms at ≥18 years of age)
- Stage 1 or stage 2 HD, defined by a United Huntington’s Disease Rating Scale-Total Functional Capacity score ≥7 at screening
- UHDRS-IS score ≤90% at screening
- UHDRS-TMS score ≥20 at screening
- Met all criteria required for randomization authorization flow and was considered eligible by the randomization authorization flow reviewer
- Willingness to comply with contraceptive requirements:
  - Female participants of childbearing potential must have had a negative β-human chorionic gonadotropin test at screening and baseline, or must have been sterile or post-menopausal.
  - Female participants with potentially fertile male partners must have used highly effective birth control methods stable for at least 3 months before screening, during the study and for 30 days after study drug discontinuation.
  - Male participants must have been sterile or used effective birth control with female partners throughout the study and for 90 days after discontinuation.
- Allowed psychotropic medication dosing (for example, antipsychotics, antidepressants) must have been stable for at least 4 weeks before baseline and throughout the study, unless clinically necessary to change.
- Allowed concomitant medication dosing must have been stable for at least 4 weeks before baseline (amiodarone must not have been used within 6 weeks of baseline).
- Capable of providing signed informed consent and complying with study requirements.

#### Exclusion criteria

Participants were excluded if any of the following applied:

- Q-TcF interval >450 ms for males or >470 ms for females at screening
- Clinically significant heart disease within 12 weeks before randomization, including:
  - History of arrhythmia, symptomatic or uncontrolled atrial fibrillation, confirmed ventricular tachycardia or left bundle branch block
  - Known congenital long Q-T syndrome or family history of same
  - Clinically significant bradycardia, sick sinus syndrome, atrioventricular block, congestive heart failure or electrolyte disturbances (hypocalcemia, hypokalemia, hypomagnesemia)
- History of epilepsy or seizures within the past 5 years
- Serious medical illness including:
  - Uncontrolled hypertension
  - Severe asthma
  - Severe hepatic disease (hepatitis B virus, hepatitis C virus, human immunodeficiency virus)
  - Severe renal disease or AIDS
  - Unstable psychiatric or neurological disorders
  - Metastatic cancer
- Known intracranial neoplasms, vascular malformations, cerebrovascular accident or intracranial hemorrhage
- Pregnancy, planning pregnancy or breastfeeding
- Use of medications that prolong Q-T interval within 4 weeks of baseline (amiodarone prohibited within 6 weeks)
- Use of nonallowed antipsychotics, tricyclic antidepressants or Class I antiarrhythmics within 4 weeks of baseline
- Use of pridopidine within 12 months before baseline
- Treatment with any investigational product within 6 weeks (or five half-lives, whichever is longer) before screening
- Receipt of gene therapy at any time
- Prior participation in any study involving tominersen
- Laboratory abnormalities at screening that were clinically significant or:
  - Creatinine clearance <30 ml min^−1^ (Cockcroft–Gault)
  - Aspartate aminotransferase or alanine aminotransferase ≥2.5× upper limit of normal
  - Gamma-glutamyl transferase ≥3.0× upper limit of normal
  - Total bilirubin >1.5 mg dl^−1^ (unless caused by Gilbert’s syndrome without liver dysfunction)
- Alcohol or substance use disorder within 6 months before screening (per Diagnostic and Statistical Manual of Mental Disorders (DSM)-5 Text Review (TR))
- Active suicidal ideation (Columbia-Suicide Severity Rating Scale score of 4 or 5) within 1 year of screening or positive suicidal behavior items, or judged to be at serious suicide risk by the Investigator
- Known allergy to any ingredient in the study drug (pridopidine, silicified microcrystalline cellulose, magnesium stearate)
- Vulnerable participants (for example, detained individuals) or those unfit because of living circumstances
- Employees or immediate family members of Sponsor, Investigator or study site staff, or those otherwise dependent.

### Participant discontinuation and withdrawal

Managing participant discontinuation and withdrawal was crucial to ensure safety, compliance and data integrity. The stopping rule specified that participants had to discontinue the study drug if their Q-TcF exceeded 500 ms or was over 480 ms with an increase of more than 60 ms from baseline (as determined by electrocardiogram). In addition, participants with CrCl <30 ml min^−1^ were required to discontinue. Additional stopping criteria included suicidal ideation (Columbia-Suicide Severity Rating Scale score ≥4 or Problem Behaviors Assessment-Short Version (PBA-s) suicidal ideation >3), seizures, symptomatic or uncontrolled atrial fibrillation, confirmed ventricular tachycardia, presence of left bundle branch block, intracranial issues, cerebrovascular events, substance use disorders or pregnancy. Other reasons included adverse events, noncompliance, consent withdrawal or study termination. Discontinued participants were encouraged to attend scheduled visits. Participants could withdraw at any time, and the reasons for withdrawal were recorded.

### Assessments and endpoints

The primary endpoint of the study was the change from baseline in the TFC score, at 65 weeks. The TFC is the standard and well-accepted clinical scale for staging and tracking the progression of HD. Scores range from 0 to 13, with 13 indicating no functional impairment and 0 representing complete incapacity. The scale assesses a participant’s ability to manage domestic chores, activities of daily living, finances, care needs and occupation. The scale is designed to detect changes in the early stages of HD (HD1 and HD2) and exhibits a floor effect in advanced stages of the disease.

The key secondary endpoint was the change in cUHDRS score from baseline at 65 weeks (ref. ^60^). The cUHDRS is a composite measure comprising four components: TFC, TMS, SDMT and SWR. These components collectively assess functional, motor and cognitive outcomes, offering a comprehensive evaluation of clinical progression in HD.

A key assumption in using the cUHDRS in clinical studies is that neuroprotective therapies targeting disease mechanisms should result in concordant improvements across all components of the cUHDRS^29,60^. To evaluate this assumption and explore the contribution of each cUHDRS component at each study visit, post hoc forest plot analyses were conducted in the mITT and PP populations off ADMs. The components were rescaled using standardization factors from Schobel’s formula (1.9, −14.9, 11.3 and 20.1 for TFC, TMS, SDMT and SWR, respectively) to visualize their relative contributions^4^.

Additional endpoints included Q-Motor measures, which provide objective, rater-independent quantifiable assessments of motor performance, in which an increase in performance indicates improvement with minimal or no placebo effects^23,55,61^. These tests evaluated motor skills essential for daily activities, including FT IOI Mean and FT IOI Standard Deviation (digitomotography), FT ITI, and Pro-Sup IOI and Pro-Sup ITI (dysdiadochomotography) measures. These assessments used precalibrated, temperature-controlled force transducers and three-dimensional position sensors to ensure high sensitivity and reliability across sessions and sites, reducing variability and placebo effects compared with clinical rating scales^55^. Data were transferred securely for centralized review and analysis was automated and blinded. FT IOI, FT ITI, Pro-Sup ITI and Pro-Sup IOI were analyzed to determine motor performance.

Cognitive performance was evaluated using the SWR and the SDMT, which assesses attention, processing speed and cognitive flexibility. The SWR evaluates attention and mental flexibility through the Stroop effect, where participants to read color names (for example, ‘red’) printed in black ink, with accuracy and speed recorded over 45 s. Higher scores reflect better performance. SWR was evaluated both as an individual cognitive measure and as a prespecified component of the composite cUHDRS endpoint. The SDMT assesses psychomotor speed and working memory by having participants match symbols to corresponding numbers within 90 s, with scores reflecting the total correct responses (maximum of 110). Both assessments were administered alongside motor tests.

TMS evaluates motor features of HD including gait, balance, oculomotor function, dysarthria, dystonia and postural stability and chorea, where lower TMS scores indicate improvement. All study raters were trained and certified through UHDRS-TMS online certification provided by the European Huntington Disease Network Motor Working group in collaboration with the ENROLL-HD study platform^55,62^.

### Statistical analysis

The primary outcome endpoint analysis was performed on the ITT population, or on the mITT population, depending on regional regulatory requirements. For the European Union (EU) regions, the primary endpoint was United Huntington’s Disease Rating Scale-Total Functional Capacity change from baseline at 65 weeks. The primary estimand was a composite of treatment policy and hypothetical strategies, defined in the ITT population, which included all randomized participants. In non-EU regions, the primary analysis was conducted using a treatment policy strategy-based estimand in the mITT population, which included all participants in the ITT population who received at least one dose of the study drug and had valid in-clinic TFC scores at both baseline and at least one post-baseline time point. Here we present all data from the mITT, which excluded nine patients from the ITT population. Sensitivity analyses were conducted on the PP population, which included participants from the mITT population who were on the study drug at week 65 (W65PP) or week 78 (W78PP), maintained compliance >80% during the study and did not have major protocol deviations impacting efficacy.

The statistical methods applied in the study, including sample size calculations, were prespecified in the protocol and detailed in the statistical analysis plan (SAP), which was finalized before treatment unblinding. The null hypothesis stated that there was no difference between pridopidine and placebo in improving TFC at week 65. A restricted maximum likelihood-based MMRM was used to analyze in-clinic observed change from baseline at each post-baseline visit based on the primary estimand. The MMRM model included fixed effects for treatment, baseline value of the endpoint, region (Europe, North America), two randomization stratification factors (antipsychotic or neuroleptic use (yes, no) and baseline HD stage (HD1, HD2)), categorical week and treatment-by-week interaction. A random intercept for participant was included. An unstructured covariance matrix was used to model within-participant correlations. Kenward–Roger approximation was used to estimate denominator degrees of freedom.

Hypothesis testing was conducted hierarchically for the primary and key secondary endpoints, maintaining a two-sided Type I error level of ≤0.05 to control for multiple comparisons. The endpoints included in the hierarchical testing sequence were:

- Change from baseline to week 65 in TFC (primary endpoint)
- Change from baseline to week 65 in cUHDRS total score (key secondary endpoint)
- Proportion of participants with improvement or no worsening (≥0-point change) at week 65 in TFC
- Change from baseline to week 52 and week 78 in TFC
- Change from baseline to week 65 in Q-Motor FT IOI Mean
- Change from baseline to week 65 in UHDRS-TMS
- Change from baseline to week 65 in SDMT
- Change from baseline to week 52 in TMS
- Proportion of participants with improvement or no worsening in Clinical Global Impression of Change at week 65.

Because the primary endpoint was not met, no further formal multiplicity adjustment was applied beyond the hierarchical testing procedure. Subgroup analyses, including those in the off-ADM population, were reported with point estimates, 95% CI and nominal *P* values, and were interpreted with appropriate caution. Prespecified sensitivity analyses included subgroup analyses of the primary endpoint (TFC) by baseline ADM status, as outlined in the SAP. Post hoc analyses were subsequently performed to evaluate cUHDRS and responder thresholds in the off-ADM subgroup.

Following the primary analysis, missing data were handled using a control-based pattern mixture model (PMM) under a Missing Not at Random (MNAR) assumption. In this approach, missing post-discontinuation outcomes were imputed based on placebo group trajectories. The assumed delta adjustment reflected a weighted estimate based on observed dropout patterns across treatment groups. Sensitivity analyses using PMM were performed for TFC, cUHDRS and other key endpoints. The results of these sensitivity analyses were consistent with the primary MMRM findings, although nominal statistical significance was not reached in the overall population.

The sample size determination aimed to ensure sufficient power to detect a statistically significant difference in change from baseline in TFC between pridopidine and placebo at week 65 at 90% power and a two-sided significance level of 0.05. Specifically, a total sample size of 372 participants provided 94% power to detect a between-group difference of 0.7 points in mean change from baseline to 65 weeks in TFC, assuming an s.d. of 1.9 and a two-tailed *t*-test. To accommodate a projected dropout rate of 22.5%, the final target sample size was set at 480 randomized participants. This dropout assumption was empirically derived from the PRIDE-HD trial^23^, in which the combined dropout rate for the placebo and pridopidine 45 mg bid groups was 22.3% among participants with baseline TFC scores between 7 and 13. Under a MNAR assumption, modeling discontinued participants to follow the trajectory of placebo after withdrawal, the estimated treatment difference of 0.565 (a weighted average of 0.7 for completers and 0.1 for dropouts) with an s.d. of 1.9 yielded 90% power to detect a statistically significant effect.

Continuous variables were summarized using descriptive statistics (mean, s.d., quartiles); categorical variables were summarized using frequency counts and percentages. Using control-based PMM, sensitivity analyses were conducted under an MNAR assumption to evaluate robustness. Continuous outcomes were analyzed using MMRM, accounting for repeated observations and assuming data were missing at random. Continuous outcomes were analyzed using MMRM, accounting for repeated observations and assuming data were missing at random. The SP included all participants who received at least one dose of study drug and was analyzed according to treatment received.

We performed a post hoc responder analysis of pridopidine’s impact on cUHDRS. However, despite the advantages of performing a responder analysis^37,63,64^, choosing a single specific cutoff to define a responder can be a limitation. To address this limitation, two complementary approaches were adopted. First, a post hoc CDF analysis was used, which does not require a specific responder cutoff but instead evaluates the entire range of responses, spanning from −10% to +10% change from baseline in cUHDRS score. The AUC difference between the pridopidine and placebo groups was assessed using a logistic model with a fixed effect for treatment. In addition, the CDFs between the treatment groups were compared nonparametrically using the KS test. Second, a specific threshold was analyzed, defined as at least a 5% improvement from baseline on the cUHDRS, a threshold considered reasonably likely to represent clinical significance given the progressive decline typically observed in HD.

All statistical tests were two-sided, with a significance threshold of 0.05. Subgroup analyses were prespecified to evaluate treatment effects by baseline HD stage, ADM use and other factors. For efficacy and safety outcomes, baseline was defined as the last nonmissing assessment before the first study drug dose. Change from baseline at scheduled visits to week 65 (weeks 26, 39, 52, 65) and to week 78 were analyzed using separate MMRM models to reduce potential bias from participants who did not reach week 78. End of study was defined as the time point when the last participant completed the week 65 visit or withdrew. All statistical analyses were conducted using SAS v.9.4.

#### Efficacy subgroup analysis

Given the potential of ADMs (antipsychotics or neuroleptics and VMAT2 inhibitors) to mask clinical measures of HD and potentially mask the efficacy outcomes of pridopidine^8,65^, subgroup analyses were conducted on participants who were off ADMs at any point during the study, including both at baseline and throughout its duration. The SAP included stratification at baseline based on antipsychotic use (yes or no) and, considering the biological plausibility that ADMs could negatively impact function and cognition in HD, subgroup analyses excluding antipsychotics and VMAT2 inhibitor use any time during the study were defined. These analyses were conducted in both the modified intent-to-treat mITT and PP populations (Fig. 1 and Table 1).

### Safety evaluation and monitoring

Participants were closely monitored throughout the study to ensure proper adherence to the dosing regimen. Adverse events were collected and reported in accordance with Good Clinical Practice guidelines. Safety monitoring included tracking the incidence of TEAEs, SAEs and any events leading to treatment and study discontinuation (Table 2; Extended Data Table 1). Investigators assessed the severity of all adverse events, and their causality to determine whether they were related to the treatment with the study drug.

An independent safety monitoring committee reviewed unblinded data during the trial to ensure participant safety. The monitoring approach ensured a comprehensive evaluation of treatment exposure, adherence and safety.

### Ethical considerations

This trial was conducted in accordance with the Declaration of Helsinki and the Council for International Organizations of Medical Sciences International Ethical Guidelines, as well as the International Council for Harmonisation guidelines for Good Clinical Practice^66^. The trial protocol and all amendments were approved by an independent review board (IRB) and independent ethics committee (IEC).

Ethical approvals were obtained from IRBs and IECs at each participating site, including but not limited to the Western Institutional Review Board (USA), Comité de Protection des Personnes Ile de France VI (France), Ethik-Kommission der Ärztekammer Hamburg (Germany), Comités de Ética de la Investigación con medicamentos (CEIm) del Hospital General Universitario Gregorio Marañón (Spain), Central Ethics Committee of the Czech Republic, Medical Ethics Committee of the University Medical Center Groningen (Netherlands) and Comitati Etici per la Sperimentazione Clinica della Regione Toscana (Italy). Written informed consent was obtained from all participants before any study-specific procedures were conducted.

The Investigator was responsible for providing written summaries of the status of the study to the IRB and IEC as required by the IRB and IEC’s policies and procedures. In addition, the Investigator notified the IRB and IEC of any SAEs or other notable safety findings as required by IRB and IEC procedures. Oversight of the study’s conduct at each site and adherence to the requirements of 21 Code of Federal Regulations (CFR), International Council for Harmonisation guidelines, the IRB and IEC and applicable local regulations were maintained. Each Investigator’s agreement to conduct and administer this study in accordance with the protocol was documented in separate study agreements with the Sponsor, as well as in other forms as required by national authorities in the country where the study center was located.

### Reporting summary

Further information on research design is available in the Nature Portfolio Reporting Summary linked to this article.

## Online content

Any methods, additional references, Nature Portfolio reporting summaries, source data, extended data, supplementary information, acknowledgements, peer review information; details of author contributions and competing interests; and statements of data and code availability are available at 10.1038/s41591-025-03920-3.

## Supplementary information

Supplementary Information Supplementary Table 1. Overview of clinical trial study sites. List of 59 international sites participating in the PROOF-HD study, including site numbers, institution names, locations, principal investigators, and sub-investigators. Each site is uniquely identified by its site number and is led by a principal investigator, with sub-investigators providing additional support where applicable. This list does not indicate authorship. Those meeting authorship criteria are named in the main manuscript author list.

Reporting Summary

## Figures and Tables

**Extended Data Table 1:**
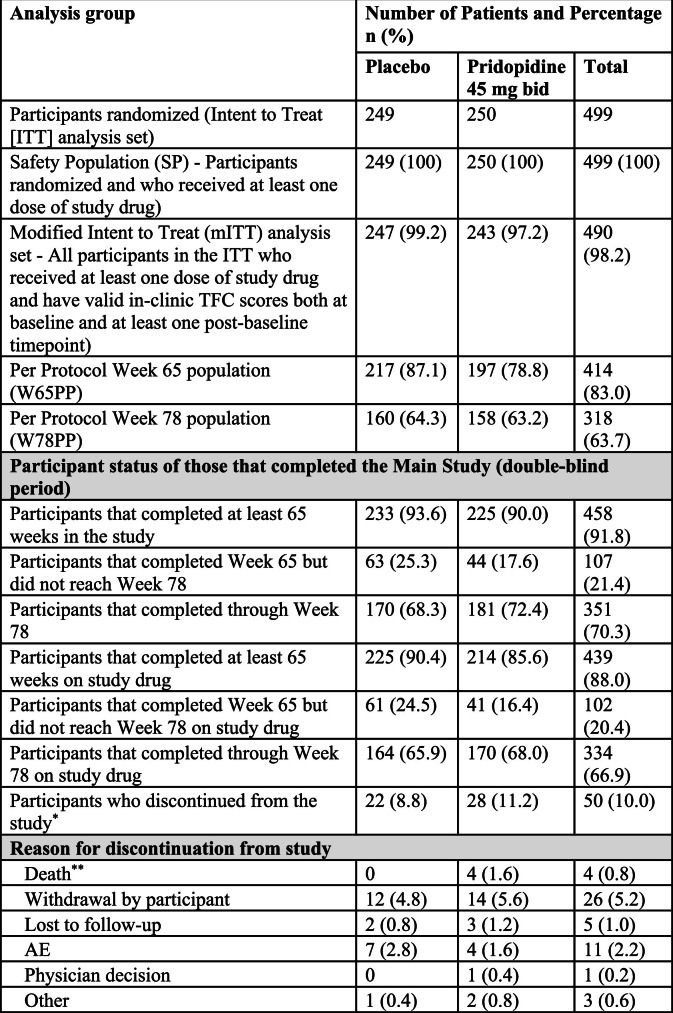
Summary of Study Treatment-Related Treatment Emergent Adverse Events

This table summarizes study treatment-related treatment emergent adverse events (TEAEs) reported in the Safety Population (SP). **Abbreviations:** bid = twice daily; TEAE = treatment emergent adverse event. **Notes:** • A TEAE is defined as an adverse event (AE) that occurred for the first time or worsened on or after initiation of treatment in the Main Study and within 14 days of stopping study treatment. • A total of 118 (23.6%) participants reported a TEAE assessed by the investigator as related to study treatment, with similar proportions in the placebo and pridopidine arms. Of these, only one participant (placebo arm) experienced a treatment-related serious adverse event (SAE). None of the treatment-related TEAEs led to death. • Study treatment-related TEAEs leading to treatment withdrawal were reported in 21 (4.2%) participants, including 9 (3.6%) in the placebo arm and 12 (4.8%) in the pridopidine arm. The majority of treatment-related TEAEs were of mild to moderate severity (114 participants, 22.8%), with similar rates between treatment arms. Severe treatment-related TEAEs were reported in 4 (0.8%) participants: Placebo arm: 1 (0.4%) participants (acute myocardial infarction); Pridopidine arm: 3 (1.2%) participants (diarrhea infectious, muscle spasms, and depression).

**Extended Data Table 2:**
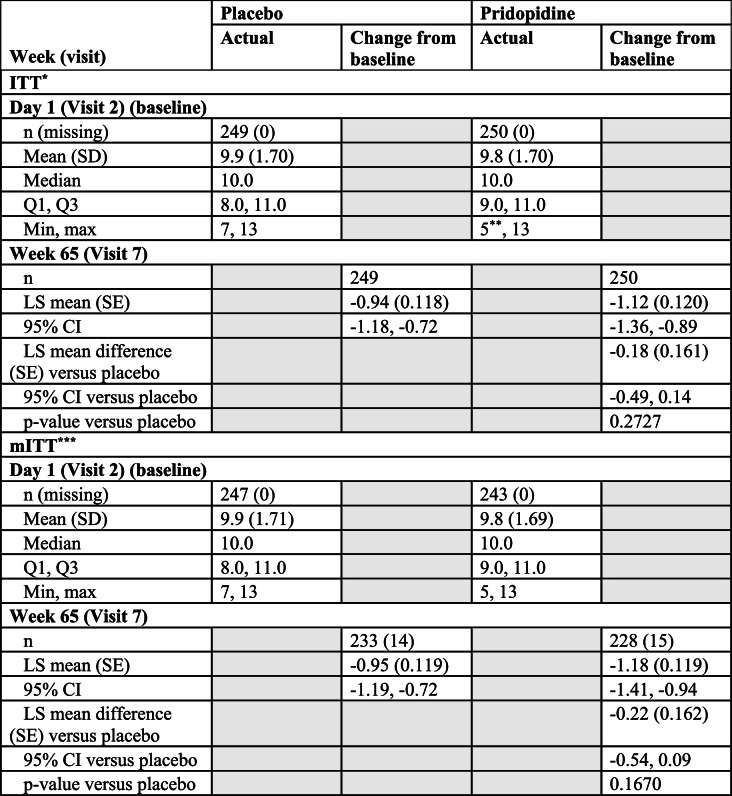
Change from Baseline in Total Functional Capacity (TFC) at Baseline and Week 65 (ITT, mITT)

This table summarizes the change from baseline in TFC for placebo and pridopidine 45 mg bid groups at baseline and Week 65 in the ITT and mITT populations. **Abbreviations:** bid = twice daily; CI = confidence interval; LS = least square; max = maximum; min = minimum; Q1, Q3 = quartile 1, 3; SD = standard deviation; SE = standard error. **Notes:** • *For ITT analysis:* Missing data were imputed using multiple imputations (MI) under the assumption of missing not at random (MNAR). Analysis and p-values for comparison of pridopidine versus placebo were generated by combining results from the mixed model for repeated measures (MMRM) for each imputed dataset. Summary statistics are not applicable due to multiple imputations. The MMRM included change from baseline in TFC score as the dependent variable and independent variables: treatment arm, baseline TFC, region, neuroleptic use or no use, baseline HD stage (HD1 and HD2), categorical week, and treatment by categorical week interaction, with Kenward-Roger approximation for degrees of freedom. An unstructured covariance matrix was used for repeated measurements at the participant level. • *For mITT analysis:* The analysis and p-values were generated using observed data in the MMRM for comparison of pridopidine versus placebo. The MMRM included change from baseline in TFC as the dependent variable and independent variables: treatment arm, baseline TFC, region, neuroleptic use or no use, baseline HD stage (HD1 and HD2), categorical week, and treatment by categorical week interaction, with Kenward-Roger approximation for degrees of freedom. An unstructured covariance matrix was used for repeated measurements at the participant level. No imputation was performed on missing data. • One participant was screened with TFC ≥ 7 but had a TFC score below 7 by the baseline visit.

**Extended Data Table 3:**
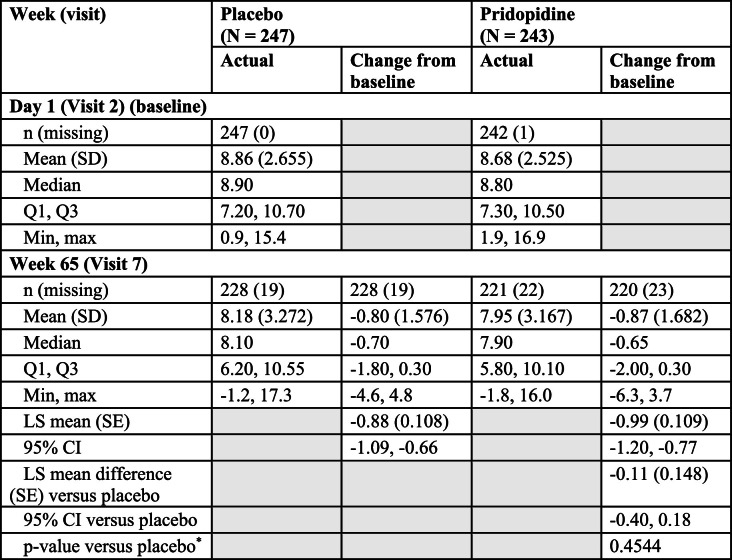
Change from Baseline to Week 65 in Composite Unified Huntington’s Disease Rating Scale (cUHDRS) Scores for Placebo and Pridopidine Groups (mITT)

This table summarizes the change from baseline to Week 65 in cUHDRS scores for placebo and pridopidine 45 mg bid groups in the mITT population. **Abbreviations:** bid = twice daily; CI = confidence interval; cUHDRS = composite Unified Huntington’s Disease Rating Scale; LS = least square; max = maximum; min = minimum; Q1, Q3 = quartile 1, 3; SD = standard deviation; SE = standard error. **Notes:** • *The p-values presented are nominal*. • Analysis and p-values were generated using observed data from a Mixed Model Repeated Measures (MMRM) for comparison of pridopidine with placebo. • In the MMRM, change in cUHDRS score from baseline was the dependent variable, and independent variables included treatment arm, baseline cUHDRS, region, neuroleptic use or no use, baseline HD stage (HD1 and HD2), categorical week, and treatment by categorical week interaction. Kenward-Roger approximation was used for degrees of freedom. • An unstructured covariance matrix was applied for repeated measurements at the participant level. No imputation was performed for missing data.

**Extended Data Table 4:**
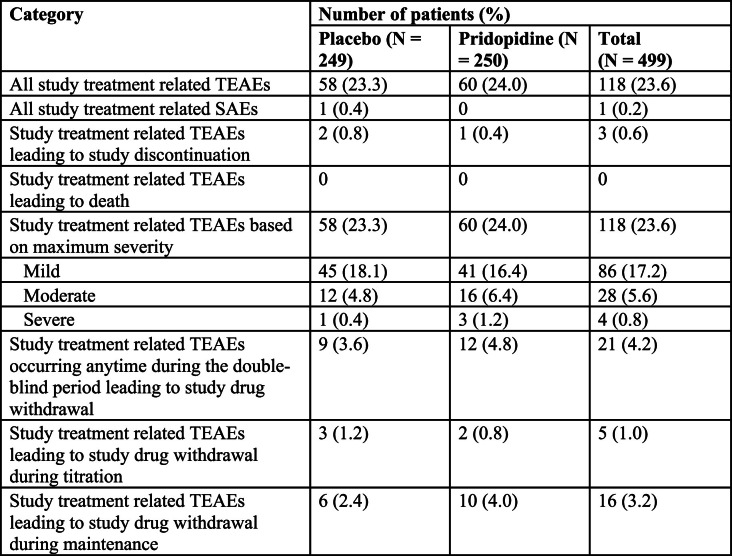
Summary of Participant Disposition (ITT)

This table summarizes the disposition of participants in the intent-to-treat (ITT) population. Percentages are based on the number of randomized participants per treatment arm. **Abbreviations:** AE = adverse event; bid = twice daily; TFC = total functional capacity. **Notes:** • Participants who discontinued from the study and study treatment are counted in both categories. • *Nine participants completed at least 65 weeks but discontinued the study. These participants are included in both the 50 who discontinued the study and the 458 who completed at least 65 weeks*. • Deaths: A total of five deaths occurred: one in the placebo arm, three in the pridopidine arm, and one in the pridopidine arm after discontinuing treatment for over a year. The latter was not deemed treatment-emergent. • The death in the placebo arm was related to a road traffic accident and categorized as a treatment-emergent adverse event (TEAE). • None of the deaths were deemed treatment-related by the investigator.

## Data Availability

The minimum dataset necessary to interpret, verify and extend the findings of this study will be made available to qualified researchers. Individual deidentified participant data (IDP), including data dictionaries, will be shared. Related documents including the study protocol, statistical analysis plan (SAP) and informed consent form template will also be available upon request and approval. Data access will be granted beginning six months after the date of publication and will remain available for a period of five years, subject to a formal request process. Access is limited to researchers affiliated with academic or nonprofit institutions and will be granted for scientifically sound and ethically approved analyses that align with the original study aims or address relevant scientific questions. Data are not deposited in a public repository due to ethical and legal constraints—including protection of participant confidentiality under applicable privacy laws (for example, GDPR). However, a redacted version of the protocol and SAP are available at https://ghi-muenster.de/protocols/proof-hd and https://ghi-muenster.de/protocols/proof-hd-sap. Requests for access to clinical trial data should be directed to the Sponsor, Prilenia Therapeutics, via email at: info@prilenia.com. Each request will be reviewed by the study sponsor or its designated data access committee, and decisions will be provided within 90 days of receipt. Approved requesters must enter into a Data Use Agreement (DUA) that stipulates: (1) no attempts to re-identify participants; (2) no unauthorized downstream sharing of data; (3) compliance with agreed-upon research purposes aligned with the original study aims or relevant scientific questions; and (4) authorship or acknowledgment requirements, consistent with the principles outlined in the International Committee of Medical Journal Editors (ICMJE) Recommendations (2024). A copy of the DUA template may be made available to requesters or to journal editors upon request. No third-party proprietary datasets were used in this study. All data were collected and analyzed by the study investigators and Sponsor as detailed in Methods.

## Code availability

No custom code was developed or used in this study.
