# Supplementary material for: Pridopidine in early-stage manifest Huntington’s disease: a phase 3 trial
Source: Nat Med. 2025 Sep 5;31(11):3780–9. doi: 10.1038/s41591-025-03920-3 (PMC12618238; doi:10.1038/s41591-025-03920-3)
Supplement: Supplementary file 1 — Supplementary Table 1. Overview of clinical trial study sites. List of 59 international sites participating in the PROOF-HD study, including site numbers, institution names, locations, principal investigators, and sub-investigators. Each site is uniquely identified by its site number and is led by a principal investigator, with sub-investigators providing additional support where applicable. This list does not indicate authorship. Those meeting authorship criteria are named in the main manuscript author list. [file 41591_2025_3920_MOESM1_ESM.pdf]

nature medicine

Supplementary information

https://doi.org/10.1038/s41591-025-03920-3

Pridopidine in early-stage manifest Huntington's disease: a phase 3 trial

In the format provided by the authors and unedited

Site Number	Institute Name and Location	Principal Investigator	Sub-Investigators/Site Coordinators
**2**	Columbia University Medical Center, New York, NY, USA	Karen Marder, MD	Radhika Jagannathan, MD<br>Sarah O'Shea, MD<br>Site coordinator(s): Jasim Uddin
**6**	Oregon Health & Site Coordinator(s)ience University, Portland, OR, USA	Amie Hiller, MD	Lauren Talman, MD<br>Site coordinator(s): Emily Leonard
**17**	Massachusetts General Hospital, Charlestown, MA, USA	H. Diana Rosas, MD	Site coordinator(s): Hannah Robins
**18**	University of Pennsylvania, Philadelphia, PA, USA	Aaron Lasker, MD	Site coordinator(s): Jennifer Klapper
**19**	University of South Florida, Tampa, FL, USA	Stephen Aradi, MD	Juan Sanchez-Ramos, MD, PhD<br>Site coordinator(s): Youshra Ahmed<br>Kristyn Kuzianik
**20**	Ohio State University, Columbus, OH, USA	Clarisse Goas, MD	Soumya Bouchachi, MD<br>Site coordinator(s): Kaleigh Clevenger<br>Nicole Vrettos
**27**	Washington University, St. Louis, MO, USA	Joel Perlmutter, MD	Site coordinator(s): Melissa Ammel<br>Matthew Lewis
**28**	Johns Hopkins University, Baltimore, MD, USA	Jee Bang, MD	Frederick Nucifora, DO, PhD; Christopher Ross, MD, PhD<br>Site coordinator(s): Etta Bernard<br>Mollie Jenckes, RN
**29**	University of Kansas Medical Center, Kansas City, KS, USA	Richard Dubinsky, MD	Site coordinator(s): Audrey Davis<br>Cameron Zoraghchi
**31**	Vanderbilt University Medical Center, Nashville, TN, USA	Daniel Claassen, MD	Amy Wynn, NP-C; James Eaton, MD<br>Site coordinator(s): Danielle Buchanan
**32**	Emory University Site Coordinator(s)hool of Medicine, Atlanta, GA, USA	Stewart Factor, MD	Laura Site Coordinator(s)orr, MD<br>Site coordinator(s): Elaine Sperin
**37**	Albany Medical College, Albany, NY, USA	Era Hanspal, MD	Eric Molho, MD<br>Site coordinator(s): Alicia Leader
**51**	University of California, San Diego, La Jolla, CA, USA	Jody Corey-Bloom, MD	Brenton Wright, MD<br>Site coordinator(s): Andrew Hall
**61**	University of California, Davis Medical Center, Sacramento, CA, USA	Vicki Wheelock, MD	Alexandra Duffy, DO<br>Site coordinator(s): Rebecca Craig<br>Kristin Phillips<br>Fernando Rodriguez
**76**	Beth Israel Deaconess Medical Center, Boston, MA, USA	Samuel Frank, MD	Simon Laganiere, MD; Daniel Press, MD<br>Site coordinator(s): Aine Russell<br>Clementina Ullman

**83**	Hereditary Neurological Disease Centre, Wichita, KS, USA	William Mallonee, MD	Bryon McNeil, MD<br>Site coordinator(s): Norberta Robertson<br>Greg Suter
**87**	University of Louisville, Louisville, KY, USA	Peter Hedera, MD	Laura Dixon, APRN; Victoria Holiday, MD<br>Site coordinator(s): Annette Robinson, RN<br>Angela Siegwald
**88**	Northwestern University Medical Center, Chicago, IL, USA	Danny Bega, MD	Danielle Larson, MD; Neil Shetty, MD<br>Site coordinator(s): Zsa Zsa Brown<br>Cynthia Poon
**89**	University of Cincinnati, Cincinnati, OH, USA	Andrew Duker, MD	Site coordinator(s): Sadie Caldwell
**96**	University of Washington and VA Puget Sound, Seattle, WA, USA	Anny Lin, MD	Ali Samii, MD<br>Site coordinator(s): Debra Del Castillo<br>Bianca Le
**119**	Duke University Medical Center, Durham, NC, USA	Burton Site Coordinator(s)ott, MD	Kathryn Moore, MD<br>Site coordinator(s): Lisa Gauger<br>Karen White-Tong
**160**	University of Florida, Gainesville, FL, USA	Nikolaus McFarland, MD	Site coordinator(s): Kyle Rizer
**199**	University of Texas Houston Medical Site Coordinator(s)hool, Houston, TX, USA	Erin Furr Stimming, MD	David Hunter, MD<br>Site coordinator(s): Brittany Duncan<br>Jamie Sims
**326**	Virginia Commonwealth University, Richmond, VA, USA	Matthew Barrett, MD	Stephaie Bissonnette, DO, MPH<br>Site coordinator(s): Ginger Norris
**333**	Georgetown University, Washington, DC, USA	Karen Anderson, MD	Site coordinator(s): Erin Koppel<br>Robin Kuprewicz<br>Mara McCartin
**343**	Rocky Mountain Movement Disorders Center, Englewood, CO, USA	Vicki Segro, MD	Rajeev Kumar, MD<br>Site coordinator(s): Jessica Crall<br>Liza Heap<br>Melanie Patton
**30**	University of Calgary, Calgary, AB, Canada	Justyna Sarna, MD	
**48**	University of British Columbia, Vancouver, BC, Canada	Lynn Raymond, MD	Blair Leavitt, MD
**98**	CHUM - Hospital Notre-Dame, Montreal, QC, Canada	Alby Richard, MD	Sylvain Chouinard, MD
**232**	True North Clinical Research, Halifax, NS, Canada	Kerrie Site Coordinator(s)hoffer, MD	Fred Archibald, MD; Rodney Brittain, MD; Ronald Wojcik, MD
**291**	Innsbruck Medical University, Innsbruck, Austria	Klaus Seppi, MD	Federico Carbone, MD; Atbin Djamshidian, MD

**388**	General University Hospital, Prague, Czech Republic	Jiri Klempir, MD	
**384**	Hopitaux Universitaires de Marseille Timone, Marseille, France	Jean-Philippe Azulay, MD	Frederique Fluchere, MD; Stephan Grimaldi, MD
**385**	Les Hospitaux d'Amiens et de Lille, Lille Cedex, France	Clemence Simonin, MD	Anne Sophie Blaise, MD; Guillaume Carey, MD
**392**	Hopital Henri Mondor, Paris, France	Anne-Catherine Bachoud-Levi, MD	Celine Joannet, MD; Lydie Lim, MD
**175**	University of Ulm, Ulm, Germany	Katrin Lindenberg, MD	Jan Lewerenz, MD; Daniel Rapp, MD
**234**	University of Aachen, Aachen, Germany	Kathrin Reetz, MD	Julia Bungenburg, MD; Stella Andrea Glasmacher, MD
**292**	Klinik Taufkirchen, Taufkirchen, Germany	Alzbeta Muehlbaeck, MD	Tetyana Blinder, MD; Rainer Hoffmann, MD
**376**	George Huntington Institute, Muenster, Germany	Stefan Bohlena, MD	Anabel Rusenberg, MD
**377**	University of Luebeck, Luebeck, Germany	Alexander Muenchau, MD	Sebastian Loens, MD; Vera Tadic, MD
**379**	Ruhr University, Bochum, Germany	Carsten Saft, MD	Jannis Achenbach, MD; Sarah von Hein, MD; Julia Jessen, MD
**228**	Casa Sollievo Della Sofferenza, San Giovanni Rotondo, Italy	Ferdinando Squitieri, MD	Sabrina Maffi, MD
**239**	Fondazione IRCCS - Instituto Neurologico Carlo Besta, Milano, Italy	Caterina Mariotti, MD	Anna Castaldo, MD
**249**	Universita di Bari Policlinico, Bari, Italy	Marina De Tommaso, MD	Conca Guilana, MD
**393**	Universita di Napoli Federico II, Napoli, Italy	FranceSite Coordinator(s)o Sacca, MD	Cinza Valeria Russo, MD
**394**	University of Bologna, Bologna, Italy	Pietro Cortelli, MD	Giorga Andrini, MD; Giovanna de Marco, MD
**44**	Leiden University Medical Center, Leiden, Netherlands	Susanne de Bot, MD	Stephanie Feleus, MD; Nadine van de Zande, MD
**387**	Maastricht University Medical Center, Maastricht, Netherlands	Mayke Oosterloo, MD	David Linden, MD

**244**	Krakow Academy of Neurology, Krakow, Poland	Monika Rudzinska, MD	Magdalena Doregowska, MD; Natalia Grabska, MD
**246**	Warsaw-IPIN Institute of Psychiatry and Neurology, Warsaw, Poland	Grzegorz Witkowski, MD	Szymion Owsiak, MD; Iwona Stepniak, MD
**386**	Szpital Specjalistyczny Swietego Wojciecha, Gdansk, Poland	Jaroslaw Slawek, MD	Magdalena Kwasniak-Butowska, MD
**176**	Hospital Ramon y Cajal, Madrid, Spain	Jose Lopez-Sendon, MD	Paula Perez, MD
**380**	Barcelona-Santa Creu i Sant Pau, Barcelona, Spain	Jaime Kulisevsky, MD	Ignacio Aracil, MD; Saul Indram, MD
**381**	Burgos Foundation, Burgos, Spain	Esther Cubo, MD	Fernando Vazquez, MD
**382**	Fundacion Hospital Universitario La Fe, Valencia, Spain	Carmen Peiro, MD	
**180**	Clinical Genetics Centre, Aberdeen, UK	Zosia Miedzybrodzka, MD	Jamie Campbell, MD; Hannah Massey, MD
**378**	Newcastle upon Tyne Hospitals, Newcastle upon Tyne, UK	Suresh Komati, MD	Khalil Memon, MD
**390**	University of Cardiff, Cardiff, UK	Duncan McLauchlan, MD	Sai Ambati, MD; Abuzer Hanif, MD
**391**	University of Birmingham, Birmingham, UK	Hugh Rickards, MD	Sangeeta Scotton, MD
