# Supplementary material for: Pridopidine in early-stage manifest Huntington’s disease: a phase 3 trial
Source: Nat Med. 2025 Sep 5;31(11):3780–9. doi: 10.1038/s41591-025-03920-3 (PMC12618238; doi:10.1038/s41591-025-03920-3)
Supplement: Supplementary file 2 — Reporting Summary [file 41591_2025_3920_MOESM2_ESM.pdf]

nature portfolio

Corresponding author(s): Ralf Reilmann
Andrew M. Tan

Last updated by author(s): Jul 17, 2025

Reporting Summary

Nature Portfolio wishes to improve the reproducibility of the work that we publish. This form provides structure for consistency and transparency in reporting. For further information on Nature Portfolio policies, see our Editorial Policies and the Editorial Policy Checklist.

Statistics

For all statistical analyses, confirm that the following items are present in the figure legend, table legend, main text, or Methods section.

n/a	Confirmed	
☐	☒	The exact sample size (*n*) for each experimental group/condition, given as a discrete number and unit of measurement
☐	☒	A statement on whether measurements were taken from distinct samples or whether the same sample was measured repeatedly
☐	☒	The statistical test(s) used AND whether they are one- or two-sided<br>*Only common tests should be described solely by name; describe more complex techniques in the Methods section.*
☐	☒	A description of all covariates tested
☐	☒	A description of any assumptions or corrections, such as tests of normality and adjustment for multiple comparisons
☐	☒	A full description of the statistical parameters including central tendency (e.g. means) or other basic estimates (e.g. regression coefficient) AND variation (e.g. standard deviation) or associated estimates of uncertainty (e.g. confidence intervals)
☐	☒	For null hypothesis testing, the test statistic (e.g. *F*, *t*, *r*) with confidence intervals, effect sizes, degrees of freedom and *P* value noted<br>*Give P values as exact values whenever suitable.*
☒	☐	For Bayesian analysis, information on the choice of priors and Markov chain Monte Carlo settings
☐	☒	For hierarchical and complex designs, identification of the appropriate level for tests and full reporting of outcomes
☒	☐	Estimates of effect sizes (e.g. Cohen's *d*, Pearson's *r*), indicating how they were calculated

Our web collection on statistics for biologists contains articles on many of the points above.

Software and code

Policy information about availability of computer code

Data collection	N/a — No custom software used for data collection. Data were collected using standard clinical assessments and validated instruments (e.g., TFC, cUHDRS, UHDRS components, Q-Motor system)
Data analysis	N/A — No custom or unpublished software was used. Data analyses were conducted using standard statistical methods (e.g., MMRM, multiple imputation) implemented in validated, commercially available software (SAS® version 9.4).

For manuscripts utilizing custom algorithms or software that are central to the research but not yet described in published literature, software must be made available to editors and reviewers. We strongly encourage code deposition in a community repository (e.g. GitHub). See the Nature Portfolio guidelines for submitting code & software for further information.

Data

Policy information about availability of data

All manuscripts must include a data availability statement. This statement should provide the following information, where applicable:

- Accession codes, unique identifiers, or web links for publicly available datasets
- A description of any restrictions on data availability
- For clinical datasets or third party data, please ensure that the statement adheres to our policy

The minimum dataset necessary to interpret, verify, and extend the findings of this study will be made available to qualified researchers. Individual de-identified participant data (IDP), including data dictionaries, will be shared. Related documents including the study protocol, statistical analysis plan (SAP), and informed

consent form template will also be available upon request and approval. Data access will be granted beginning six months after the date of publication and will remain available for a period of five years, subject to a formal request process. Access is limited to researchers affiliated with academic or non-profit institutions and will be granted for scientifically sound and ethically approved analyses that align with the original study aims or address relevant scientific questions.

Data are not deposited in a public repository due to ethical and legal constraints—including protection of participant confidentiality under applicable privacy laws (e.g., GDPR). However, a redacted version of the protocol and SAP are available at https://ghi-muenster.de/protocols/proof-hd and https://ghi-muenster.de/protocols/proof-hd-sap.

Requests for access to clinical trial data should be directed to the Sponsor, Prilenia Therapeutics, via email at: info@prilenia.com. Each request will be reviewed by the study sponsor or its designated data access committee, and decisions will be provided within 90 days of receipt.

Approved requesters must enter into a Data Use Agreement (DUA) that stipulates:
• No attempts to re-identify participants;
• No unauthorized downstream sharing of data;
• Compliance with agreed-upon research purposes aligned with the original study aims or relevant scientific questions;
• Authorship or acknowledgment requirements, consistent with the principles outlined in the International Committee of Medical Journal Editors (ICMJE) Recommendations (2024)

A copy of the DUA template may be made available to requesters or to journal editors upon request. No third-party proprietary datasets were used in this study. All data were collected and analyzed by the study investigators and Sponsor as detailed in the Methods section.

Research involving human participants, their data, or biological material

Policy information about studies with human participants or human data. See also policy information about sex, gender (identity/presentation), and sexual orientation and race, ethnicity and racism.

Reporting on sex and gender

Sex was self-reported by participants at screening and recorded in the electronic case report forms (eCRFs). Gender identity was not specifically assessed. Randomization and statistical analyses were not stratified by sex or gender, and these characteristics were not used as inclusion or exclusion criteria. The study was not powered to detect sex- or gender-based differences.

Reporting on race, ethnicity, or other socially relevant groupings

Race and ethnicity were not systematically collected or analyzed in this study. The trial enrolled participants across 12 countries in North America and Europe, and geographic region was included as a covariate in the primary statistical model. Socially relevant groupings beyond geographic region were not used for eligibility criteria, stratification, or subgroup analyses.

Population characteristics

The study enrolled 499 adults (≥25 years old) with early-stage manifest Huntington's disease (TFC 7–13), confirmed by ≥36 CAG repeats in the HTT gene. Participants were recruited from 59 clinical sites across 12 countries in North America and Europe. Both male and female participants were eligible; sex was self-reported at screening. The population was clinically homogeneous by HD stage, but no restrictions were placed on race, ethnicity, or socioeconomic status.

Recruitment

Participants were recruited from 59 clinical trial sites across 12 countries in North America and Europe between October 2020 and March 2023. Recruitment was conducted through physician referrals, site outreach, and registries of individuals with Huntington's disease. As with most clinical trials, some degree of self-selection bias may be present, as participants willing to enroll in research may differ from the general HD population in motivation, access to care, or health literacy. However, broad geographic distribution and inclusive criteria were intended to support generalizability within early-stage HD. No stratification or enrollment targeting was based on sex, race, or socioeconomic status.

Ethics oversight

Ethics approval was obtained from independent ethics committees or institutional review boards (IRBs) at each participating site, including the Western Institutional Review Board (USA), Comité de Protection des Personnes Ile de France VI (France), Ethik-Kommission der Ärztekammer Hamburg (Germany), and others as detailed in the manuscript. The study was conducted in accordance with the Declaration of Helsinki, ICH Good Clinical Practice (GCP) guidelines, and applicable regulatory requirements. The trial was registered at ClinicalTrials.gov (NCT04556656) and EudraCT (2020-002822-10). Written informed consent was obtained from all participants prior to any study procedures.

Note that full information on the approval of the study protocol must also be provided in the manuscript.

Field-specific reporting

Please select the one below that is the best fit for your research. If you are not sure, read the appropriate sections before making your selection.

☒ Life sciences ☐ Behavioural & social sciences ☐ Ecological, evolutionary & environmental sciences

For a reference copy of the document with all sections, see nature.com/documents/nr-reporting-summary-flat.pdf

Life sciences study design

All studies must disclose on these points even when the disclosure is negative.

Sample size	A total of 499 participants were randomized 1:1 to receive pridopidine or placebo. Sample size was determined based on statistical power calculations to detect differences in Total Functional Capacity (TFC) decline, accounting for expected dropout and disease progression rates.
Data exclusions	Data exclusions were prespecified. Participants without any post-baseline efficacy data were excluded from the mITT population (n=10 exclusions: 3 pridopidine, 7 placebo). Major protocol deviations led to exclusions from the PP population. No ad hoc exclusions were made.
Replication	n/a - This was a single, prospective, multicenter randomized clinical trial.
Randomization	Participants were randomized 1:1 to pridopidine or placebo using a centralized, Sponsor-generated sequence. Randomization was stratified by HD stage (HD1 vs HD2) and baseline antipsychotic use (yes/no). Allocation was concealed from investigators and participants.
Blinding	This was a double-blind study. Participants, investigators, site staff, and outcome assessors were blinded to treatment assignment throughout the 78-week study period, including the extension phase. Matching placebo capsules were indistinguishable from active drug.

Reporting for specific materials, systems and methods

We require information from authors about some types of materials, experimental systems and methods used in many studies. Here, indicate whether each material, system or method listed is relevant to your study. If you are not sure if a list item applies to your research, read the appropriate section before selecting a response.

Materials & experimental systems

n/a	Involved in the study	
☒	☐	Antibodies
☒	☐	Eukaryotic cell lines
☒	☐	Palaeontology and archaeology
☒	☐	Animals and other organisms
☐	☒	Clinical data
☒	☐	Dual use research of concern
☒	☐	Plants

Methods

n/a	Involved in the study	
☒	☐	ChIP-seq
☒	☐	Flow cytometry
☒	☐	MRI-based neuroimaging

Clinical data

Policy information about clinical studies

All manuscripts should comply with the ICMJE guidelines for publication of clinical research and a completed CONSORT checklist must be included with all submissions.

Clinical trial registration	ClinicalTrials.gov (NCT04556656) and EudraCT (2020-002822-10)
Study protocol	The full trial protocol and statistical analysis plan (SAP) will be made available via a publicly accessible portal. A redacted version of the protocol is available at https://ghi-muenster.de/protocols/proof-hd, and a redacted version of the SAP is available at https://ghi-muenster.de/protocols/proof-hd-sap. For submission for peer review, full SAP and protocols accompany the manuscript as separate documents.
Data collection	Data collection occurred from October 23, 2020 (first participant enrollment) through March 14, 2023 (last participant last visit).
Outcomes	The predefined primary outcome was the change from baseline to Week 65 in the Total Functional Capacity (TFC) score, a validated clinical scale assessing functional abilities in Huntington's disease. The predefined key secondary outcome was the change from baseline to Week 65 in the composite Unified Huntington's Disease Rating Scale (cUHDRS) score, which combines assessments of functional capacity (TFC), motor function (Total Motor Score [TMS]), and cognitive performance (Symbol Digit Modalities Test [SDMT] and Stroop Word Reading [SWR]).<br><br>All outcomes were assessed during in-person study visits using validated tools administered by trained and certified clinical raters. Motor function was also evaluated using Q-Motor assessments, which provide objective, automated, and rater-independent measurements to minimize variability and enhance sensitivity.

Plants

Seed stocks	n/a
Novel plant genotypes	n/a
Authentication	n/a
